# Gut microbiota dysbiosis and metabolic alterations in rheumatoid arthritis: a barrier to periodontal repair

**DOI:** 10.1136/rmdopen-2026-006931

**Published:** 2026-05-21

**Authors:** Di Cui, Yingying Zhou, Yibing Zhou, Ruiyang Ge, Haowei Mao, Motilal Mathesh, Lei Han, Wenrong Yang, Fuhua Yan

**Affiliations:** 1 Nanjing Stomatological Hospital, Affiliated Hospital of Medical School, Institute of Stomatology, Nanjing University, Nanjing, China; 2 School of Life and Environmental Sciences, Centre for Sustainable Bioproducts, Deakin University, Melbourne, Victoria, Australia

**Keywords:** Rheumatoid Arthritis, Bone Density, Inflammation, Risk Factors

## Abstract

**Objective:**

To investigate the impact of rheumatoid arthritis (RA) on periodontal healing and the underlying mechanisms.

**Methods:**

Mandibular periodontal bone defect (PBD) and collagen-induced arthritis (CIA) models were established in male Sprague-Dawley rats aged 6 weeks, assigned to four groups: control (CON), CIA, PBD and CIA+PBD. Periodontal repair was evaluated at 1, 3 and 6 weeks. To examine the contribution of gut microbiota, pseudo-germ-free rats with PBD received 3-week faecal microbiota transplantation (FMT) from either healthy or CIA donors. Arthritis severity was assessed by paw thickness and arthritis index, while bone microarchitecture was examined by micro-CT and histology. Gut microbiota and metabolites were analysed using 16S ribosomal RNA high-throughput sequencing and untargeted metabolomics.

**Results:**

CIA was found to significantly impair periodontal bone healing and suppress osteogenesis-related markers, including runt-related transcription factor 2 and alkaline phosphatase. Compared with CON rats, CIA and PBD, CIA+PBD groups exhibited gut microbial dysbiosis and metabolic alterations, particularly in arachidonic acid and tryptophan pathways. FMT from CIA donors further increased osteoclast numbers and delayed bone regeneration. Furthermore, gut-derived factors from CIA animals were associated with increased macrophage expression of pro-inflammatory cytokines, including tumour necrosis factor-alpha and interleukin-1 beta.

**Conclusion:**

Overall, RA-related gut microbiota dysbiosis and metabolic disturbances are linked to impaired periodontal healing, potentially through enhanced inflammatory responses. This study highlights a microbiome-immune-metabolic axis that may influence periodontal regeneration in RA.

**WHAT IS ALREADY KNOWN ON THIS TOPIC**

- Rheumatoid arthritis (RA) and periodontitis are closely associated chronic inflammatory diseases, and increasing evidence suggests a role for gut microbiota in systemic immune regulation.
- Previous studies have mainly focused on how periodontitis influences RA, while the impact of RA on periodontal tissue repair remains poorly understood.
- In addition, the contribution of gut microbiota to this interaction has not been fully elucidated.

**WHAT THIS STUDY ADDS**

- This study shows that gut microbial dysbiosis and metabolic alterations contributed to the impact effects of RA on periodontal bone regeneration.
- Using faecal microbiota transplantation and in vitro macrophage stimulation assays, we found that gut-derived factors are associated with increased inflammatory responses.
- These findings suggest a coordinated microbiome-immune-metabolic axis linking RA to impaired periodontal healing.

**HOW THIS STUDY MIGHT AFFECT RESEARCH, PRACTICE OR POLICY**

- These findings highlight the importance of considering systemic conditions in RA and gut microbiota status in periodontal regeneration.
- Future research should focus on validating the underlying mechanisms through targeted functional approaches.
- Such efforts may help improve therapeutic strategies for patients with RA and periodontal disease.

## Introduction

Periodontitis is a chronic, multifactorial, biofilm-induced inflammatory disease of the periodontium.^1^ Global burden of disease analyses indicate that severe periodontitis is the sixth most prevalent condition worldwide, affecting approximately 11% of adults.^2^ As the leading cause of tooth loss in adults, periodontitis is also linked to various systemic conditions, including diabetes, cardiovascular disease, Alzheimer’s disease and rheumatoid arthritis (RA),^3^ through multifactorial mechanisms.^1 4 5^

RA is a systemic autoimmune disease driven by genetic and environmental factors, and characterised by persistent synovial inflammation, synovial hyperplasia, progressive multiarticular cartilage and bone destruction and the production of autoantibodies, including rheumatoid factor and anticitrullinated protein antibodies, with 0.5%–1.0% global prevalence.^6^ Growing evidence suggests a strong association between periodontitis and RA, with significant correlations in prevalence and disease severity.^7^ These conditions share chronic inflammatory features, destructive bone remodelling and common risk factors such as human leucocyte antigen DR beta 1 gene genetic susceptibility, smoking and ageing. The two diseases exhibit similar pathogenic pathways, where activation of innate and adaptive immunity drives the release of pro-inflammatory cytokines, such as tumour necrosis factor-alpha (TNF-α), interleukin-1 beta (IL-1β) and interleukin-6 (IL-6), leading to sustained inflammation, connective tissue breakdown and bone erosion. The periodontal pathogen *Porphyromonas gingivalis* expresses bacterial peptidyl deaminase, which induces protein citrullination and subsequent autoantibody formation, contributing to the onset and progression of RA.^8^ It has been reported that RA disease activity (Disease Activity Score in 28 joints using C reactive protein score) correlates with periodontitis severity, with moderate-to-severe periodontal disease (stage II–IV) more common in patients with high RA activity.^9^ Compared with healthy individuals, patients with RA also demonstrate more severe symptoms, such as greater clinical attachment loss, probing depth, bleeding on probing values, increased tooth loss and alveolar bone resorption.^10^ Animal experiments further show that mice with a collagen-induced arthritis (CIA) model exhibit significantly more periodontal destruction, including alveolar bone loss, than the control (CON) group.^11^ Moreover, CIA elevates inflammatory cytokines (TNF-α, IL-1β, IL-6 and interleukin-17) in periodontal tissues, enhances osteoclast activity, thereby initiating or exacerbating alveolar bone loss,^11^ which can be mitigated by oral antiseptics or antibacterial agents.^12^ Although it has been well established that RA exacerbates periodontal bone destruction, its impact on the subsequent healing and regenerative capacity of periodontal tissues remains largely unexplored. Future studies should therefore aim to clarify the underlying mechanisms, particularly whether systemic dysregulation, such as gut microbiota imbalance, mediates this effect.

In this context, recent work has increasingly focused on elucidating the interplay between intestinal flora and periodontal bone metabolism. Numerous studies have highlighted the critical role of the gut-alveolar axis, showing that commensal gut microbiota are essential for regulating osteoimmune responses and maintain alveolar bone homeostasis.^13 14^ When this microbial balance is disrupted, dysbiosis compromises the intestinal epithelial barrier and increases permeability, allowing intestinal pathogens to influence periodontal bone metabolism through microbial, immune, endocrine and circulatory pathways, thereby exacerbating periodontitis and alveolar bone loss.^15 16^ Conversely, interventions that restore gut microbial homeostasis, such as probiotic supplementation, have been reported to attenuate periodontal inflammation and significantly reduce alveolar bone loss in periodontitis.^14 17^ With the advent of high-throughput sequencing technologies, accumulating evidence indicates that gut microbiota dysbiosis is a key feature of RA. Metagenomic shotgun sequencing and association analyses identified both compositional and functional alterations in the gut and oral microbiomes of patients with RA, characterised by a reduction in *Haemophilus* species and an enrichment of *Lactobacillus salivarius*. Notably, these changes were reversed after treatment with disease-modifying antirheumatic drugs.^18^ Taken together, the above evidence supports the hypothesis that RA affects periodontal tissues by inducing gut microbiota dysbiosis.

In this study, we investigated whether RA impairs periodontal tissue regeneration by establishing a rat model combining CIA with experimental periodontal bone defect (PBD). To further evaluate the contribution of the gut microbiota, faecal microbiota transplantation (FMT) was performed by transferring faecal material from RA rats into recipient rats with periodontal defects. Because the PBD model represents an acute regenerative injury rather than chronic biofilm-induced periodontitis, the present study was designed to examine whether RA-associated systemic dysbiosis creates a host environment that compromises periodontal healing capacity, rather than to fully reproduce the pathogenesis of periodontitis. Overall, the results suggest that gut microbiota dysregulation and metabolic imbalance may jointly contribute to impaired periodontal repair under RA-associated systemic conditions.

## Materials and methods

### Animals

Male Sprague-Dawley rats (aged 6 weeks; 200±20 g) were obtained from Zhejiang Vital River Laboratory Animal Technology (Zhejiang, China). Animals were maintained in a specific pathogen-free facility at Nanjing Agricultural University Experimental Animal Centre under controlled conditions (22±2°C; 50±20% humidity; 12 hours light/dark cycle) with libitum access to chow and water. After a week of acclimatisation, animals were randomly assigned to four groups: the CON group, the CIA group (CIA), the PBD group (PBD), the combined CIA and PBD group (CIA+PBD). The experiment groups were subdivided by experimental duration (1, 3 or 6 weeks; n=6 each).

To investigate the mechanism by which RA influences periodontal regeneration, FMT was administered via oral gavage. Forty-two rats were randomly allocated into six groups: (1) saline-gavage for 1 week (CON-1w group); (2) antibiotic-gavage for 1 week to establish the pseudo-germ-free (PGF) model (PGF-1w group); (3) untreated controls (CON group); (4) CIA rat model (CIA group); (5) PGF rats with PBD receiving FMT from healthy control donors (FCON group) and (6) PGF rats with PBD receiving FMT from CIA donors (FCIA group). Following adaptive feeding, the CIA model was induced. Rats then received a 1-week course of oral antibiotics to establish the PGF model, after which the PBD model was established. FMT was administered orally once every other day for 3 weeks. At each experimental end point, tissue specimens were collected for subsequent analysis.

### Induction and assessment of CIA in rats

CIA was induced as previously described, with minor modifications.^19 20^ A type II collagen (CII) emulsion (1 mg/mL) was prepared by homogenising bovine CII (2 mg/mL; Chondrex, Redmond, Washington, USA; 20022) with complete Freund’s adjuvant (2 mg/mL; Chondrex, 7001) at a 1:1 (v:v) ratio at 4°C. Rats anaesthetised with 2% isoflurane received subcutaneous injections of 0.1 mL emulsion into the back, base of the tail and bilateral hind paws as the initial immunisation (day 0). On day 7, a booster injection of 0.1 mL emulsion was administered into both hind-paw pads following the same protocol. After confirming successful CIA induction, rats were monitored daily for general condition, including mental status, fur coat colour and body weight. Arthritis severity was assessed every 3 days by measuring paw swelling and calculating arthritis index (AI) scores, defined as the summation of scores from all four paws (maximum 16 points).^21^ Hind-paw thickness was measured using a vernier calliper and the paw-swelling rate was calculated as:


paw swelling (%)= (t1−t0) / t0×100%

where t0 and t1 represent mean bilateral hind-paw thickness before and after inflammation.

### Construction of PBD, PGF model and FMT

Mandibular PBDs were fabricated under isoflurane anaesthesia as previously reported.^22 23^ After shaving and disinfection, a full-thickness incision (~2 cm) was made along the inferior border of the right mandible to expose the buccal alveolar bone of the first and second molars. Under continuous saline irrigation, a 4.5×2×1 mm bone defect was prepared using a low-speed ball drill. The defect was covered with a 6×3.5 mm sterile Bio-Gide collagen membrane (Yantai Zhenghai Biotech, Yantai, China) and the incision was sutured. In the second experiment, a collagen repair material (Wuxi Biotek Biotechnology, Wuxi, China) of matched size was placed within the defect before applying the membrane.

To construct a rat dysbiosis model,^24 25^ a broad-spectrum antibiotic suspension (200 mg/mL vancomycin, 400 mg/kg ampicillin, 400 mg/kg neomycin sulfate and 400 mg/kg metronidazole) was prepared in saline. PGF-1w rats received suspension by oral gavage at 1 mL/kg once daily for 1 week, whereas CON-1w rats received an equivalent volume of saline. PGF status was assessed by 16S ribosomal RNA (rRNA) gene sequencing of caecal contents following antibiotic treatment.

To investigate the role of gut microbiota in the association between RA and periodontal regeneration, FMT was performed. For each preparation, 3 g of fresh faecal samples were collected from both CON and CIA donor rats and suspended in 15 mL of sterile 0.9% saline solution at 37°C, followed by thorough homogenisation. The suspension was centrifuged at 800 rpm for 5 min and the supernatant was collected. Each recipient rat in the FCON and FCIA groups received 1 mL of the suspension from the CON and CIA groups, respectively, by oral gavage.

### Micro-CT, histology and immunohistochemistry

Bilateral mandibles and hind limbs were harvested for micro-CT (µCT) scanning (Bruker Skyscan 1276, Bruker MicroCT, Kontich, Belgium; voxel size 18 µm) and subsequent histological and immunohistochemistry (IHC) analyses. All samples were oriented uniformly using Dataviewer software (V.1.5.4.0, Bruker, Billerica, Massachusetts, USA) and coronal jaw slices and transverse ankle-joint slices were obtained. Finally, regions of interest were selected and bone microarchitecture parameters, including bone mineral density (BMD), bone volume/total volume (BV/TV), trabecular thickness (Tb.Th) and trabecular spacing (Tb.Sp), were quantified using CTan software (V.1.5.4.0, Bruker).

Samples were fixed for 48 hours and decalcified for 2 months. After embedding, 4 µm tissue sections were prepared for H&E, safranine-O/fast green (SO-FG), tartrate-resistant acid phosphatase (TRAP) and Masson staining. Histopathological scoring was performed in a blinded manner. Synovial inflammation^26^ and bone erosion^27^ were assessed from H&E-stained ankle joints, while cartilage damage was graded semi-quantitatively using the Mankin scoring system for SO-FG staining.^28^ IHC staining was performed on mandibular sections to evaluate osteogenic markers (runt-related transcription factor 2 (Runx2) and alkaline phosphatase (ALP)), inflammatory signalling pathways (nuclear factor kappa-B (NF-ĸB)) and glycolytic activity (hexokinase 2 (HK2)). In addition, TRAP staining was used to analyse osteoclast formation and bone resorption activity in the mandible and ankle joints. Primary antibodies were purchased from Servicebio (Wuhan, China) and diluted as recommended. Negative controls were prepared by substituting primary antibodies with phosphate-buffered saline (PBS). Sections were examined microscopically and the images were acquired using a PANNORAMIC MID scanner (3DHISTECH, Budapest, Hungary). Semi-quantitative analyses were performed using Image-Pro Plus V.6.0 software (Media Cybernetics, Rockville, Maryland, USA).

### Microbiome analysis using 16S rRNA gene sequencing

#### DNA extraction, PCR amplification and Illumina NovaSeq PE250 sequencing

Microbial DNA was extracted from caecal contents using the OMEGA Soil DNA Kit (M5635-02; Omega Bio-Tek, Norcross, Georgia, USA). The V3–V4 hypervariable regions of the 16S rRNA gene were amplified using primers 338F (5’-ACTCCTACGGGAGGCAGCA-3’) and 806R (5’-GGACTACHVGGGTWTCTAAT-3’). The PCR products were separated on 2% agarose gels and the concentration and purity were assessed with a NanoDrop 2000 UV spectrophotometer (Thermo Fisher Scientific, Waltham, Massachusetts, USA). Amplicons were purified using VAHTS DNA Clean Beads (Vazyme, Nanjing, China) and quantified with the Quant-iT PicoGreen dsDNA Assay Kit (Invitrogen, Carlsbad, California, USA). Following library construction with the TruSeq Nano DNA LT Library Prep (Illumina, San Diego, California, USA), paired-end sequencing (PE250) was performed on the NovaSeq platform (Illumina).

#### Bioinformatic analysis

Assembled reads were quality-filtered by trimming barcodes and primers and retaining 16S tags between 220 and 500 bp. After calculating tag copy numbers and removing redundant sequences, only tags occurring more than once were clustered into operational taxonomic units (OTUs). Reads were ranked by abundance and OTU clustering was performed using UPARSE (http://drive5.com/uparse/), a method for generating clusters (OTUs) from next-generation sequencing reads of marker genes, at 97% similarity. Subsequent analyses, including alpha-diversity/beta-diversity and linear discriminant analysis effect size (LEfSe), were conducted using the Paisono Gene Platform (Shanghai Personal Biotechnology, Shanghai, China) with R language and Quantitative Insights Into Microbial Ecology software.^29^ Because this analysis was based on partial 16S rRNA gene sequencing (V3–V4 region), taxonomic resolution was considered most reliable at the genus level.

### Non-targeted liquid chromatography-mass spectrometry analysis of gut metabolomes

Tissue samples (100 mg) were extracted with methanol (1000 μL) containing 5 ppm 2-chlorophenylalanine, homogenised and centrifuged at 12 000 rpm. Supernatants were filtered (0.22 μm) for liquid chromatography-mass spectrometry analysis using a Thermo Ultimate 3000 system (Thermo Fisher Scientific). Quality control samples were prepared by pooling 10 μL from each extract. Metabolites were separated on a Vanquish UPLC system (Thermo Fisher Scientific) equipped with an ACQUITY UPLC HSS T3 column (2.1×100 mm, 1.8 µm; Waters, Milford, Massachusetts, USA) at 40°C, with a flow rate of 0.3 mL/min, injection volume 2 μL, under gradient elution. Detection was performed using high-resolution MS instruments (TripleTOF5600plus, SCIEX, Framingham, Massachusetts, USA and Q Exactive Focus, Thermo Fisher Scientific) in both positive and negative electrospray ionisation modes. Raw data were processed using ProteoWizard and XCMS (V.3.1.3) for peak picking, alignment and correction. Metabolites were identified using the PSNGM database (Shanghai Personal Biotechnology), mzCloud Advanced Mass Spectral Database (Thermo Fisher Scientific), LIPID MAPS (Lipid Metabolites and Pathways Strategy; University of California, San Diego, California, USA), the Human Metabolome Database (University of Alberta, Edmonton, Canada), the MassBank of North America (UC Davis, Davis, California, USA) and the NIST Tandem Mass Spectral Library (NIST_2020_MSMS; NIST, Gaithersburg, Maryland, USA). Statistical analyses included clustering with the pheatmap package and multivariate modelling (principal component analysis, partial least squares discriminant analysis, orthogonal partial least squares discriminant analysis (OPLS-DA)) using the ropls package. Differential metabolites were subjected to Kyoto Encyclopedia of Genes and Genomes enrichment using clusterProfiler (V.4.6.0). All analyses were conducted by Shanghai Personal Biotechnology.

### In vitro assays

Caecal contents from CON and CIA rats were suspended in sterile PBS (w/v=1:1), homogenised by vertexing and sequentially centrifuged. The supernatant was filtered through a 0.22 μm membrane to obtain faecal microbiota supernatant (FMS). The stock solution (FMS-100) was diluted twofold, 10-fold and 50-fold for subsequent experiments. RAW 264.7 macrophages seeded in 96-well plates (1×10⁵ cells/well) were cultured overnight. Cells were treated with FMS at different concentrations or lipopolysaccharide (LPS, 1 μg/mL) as a positive control. Cell viability was assessed using Cell Counting Kit-8 (CCK-8, Dojindo) according to the manufacturer’s instructions after incubating for 1, 1.5 and 2 hours. The FMS concentration showing noticeable suppression of cell viability was selected for subsequent assays. RAW 264.7 cells were seeded in 6-well plates (2×10⁶ cells/well) and then treated with selected concentration of FMS. Total RNA was extracted and quantitative reverse transcription-PCR was performed to measure TNF-α (F: ATGGCCTCCCTCTCATCAGT, R: TTTGCTACGACGTGGGCTAC) and IL-1β (F: GCATCCAGCTTCAAATCTCGC, R: TGTTCATCTCGGAGCCTGTAGTG) expression levels, with glyceraldehyde-3-phosphate dehydrogenase (F: GAGAGTGTTTCCTCGTCCCG, R: ATCCGTTCACACCGACCTTC) as an internal control. Relative expression was calculated using the 2^−ΔΔCt^ method.

### Statistical analysis

All statistical data are presented as mean±SD. Experiments were based on at least five independent replicates. Data analysis and graphing were performed using GraphPad Prism V.9.0 (GraphPad software, San Diego, California, USA). Normality was assessed using the Kolmogorov-Smirnov test and homogeneity of variance using Levene’s test. Comparisons between two groups were performed using two-tailed t-tests, Welch’s tests or Mann-Whitney U tests, as appropriate. For multiple-group comparisons, one-way analysis of variance followed by Tukey’s post hoc test was applied. Statistical significance was set at p<0.05.

## Results

### Establishment and validation of the CIA rat model

No mortality occurred during the study. CON rats remained healthy, whereas CIA and CIA+PBD rats developed pronounced hind-paw swelling, erythema and deformity (online supplemental figure S1A). As the disease progressed, CIA rats developed poor mental state, coarse fur and hair loss, along with reduced food and water intake, and weight loss. Furthermore, the skin around swollen paws became ulcerated, dark red and indurated, leading to restricted movement with limping and dragging. Compared with CON and PBD rats, CIA and CIA+PBD groups exhibited a substantial early decline in body weight (p<0.01), most pronounced at weeks 3–4 (vs CON, p<0.001; vs PBD, p<0.01), followed by a slight recovery (online supplemental figure S1C). Paw swelling increased considerably after the initial immunisation in CIA and CIA+PBD groups, peaking on day 17 relative to CON rats. Swelling gradually declined but remained significantly higher than in the CON group (p<0.01). The CIA+PBD group showed slightly less swelling than the CIA group, although this difference reached significance only during the peak period (days 17–24) (p<0.01) (online supplemental figure S1D). AI, obtained by measuring the degree of inflammation in the paws, was highest in CIA rats on day 28 (online supplemental figure S1E).

The µCT results showed smooth, well-defined ankle joint surfaces in CON and PBD rats, with no structural damage or irregular protrusions (online supplemental figure S1B). However, CIA and CIA+PBD rats displayed considerable bone destruction in the distal femur and tibial-talonavicular and tarsometatarsal joints, including bone erosion, blurred joint margins and joint-space loss. Bone parameters did not differ significantly between CON and PBD groups (online supplemental figure S1F-I). Compared with CON and PBD groups, CIA and CIA+PBD rats exhibited progressive reductions in BMD, BV/TV and Tb.Th, along with an increasing trend in Tb.Sp over time. The differences were most significant in weeks 3 and 6 (week 3: p<0.0001–0.01; week 6: p<0.0001–0.05). These findings confirm that CIA induces significant joint swelling and bone destruction, whereas PBD alone does not significantly influence arthritis severity.

Histological examination using HE, SO-FG and TRAP staining techniques suggested corresponding tissue alterations (online supplemental figure S2A). CON rats showed preserved joint architecture, intact synovium and well-organised chondrocytes. Conversely, rats in the CIA and CIA+PBD groups exhibited substantial ankle-joint erosion, narrowed joint spaces, cartilage fissuring, reduced matrix staining, synovial hypertrophy with disorganised cell arrangement, extensive inflammatory cell infiltration and pannus formation. Subchondral bone loss was evident, with irregular trabeculae and pronounced bone defects. Scores for synovitis, bone erosion and cartilage damage were significantly higher in CIA and CIA+PBD groups than in the CON group at weeks 3 and 6 (week 3: p<0.0001 for all scores; week 6: p<0.0001 for all scores), with no significant difference between the CIA and CIA+PBD groups (online supplemental figure S2B-D). TRAP staining further demonstrated significantly greater numbers of osteoclasts in both CIA and CIA+PBD groups than in the CON group at weeks 3 and 6 (week 3: both p<0.0001; week 6: CIA vs CON, p<0.0001; CIA+PBD vs CON, p<0.01; online supplemental figure S2E).

### RA compromises PBD healing in PBD rats

**Figure 1:**
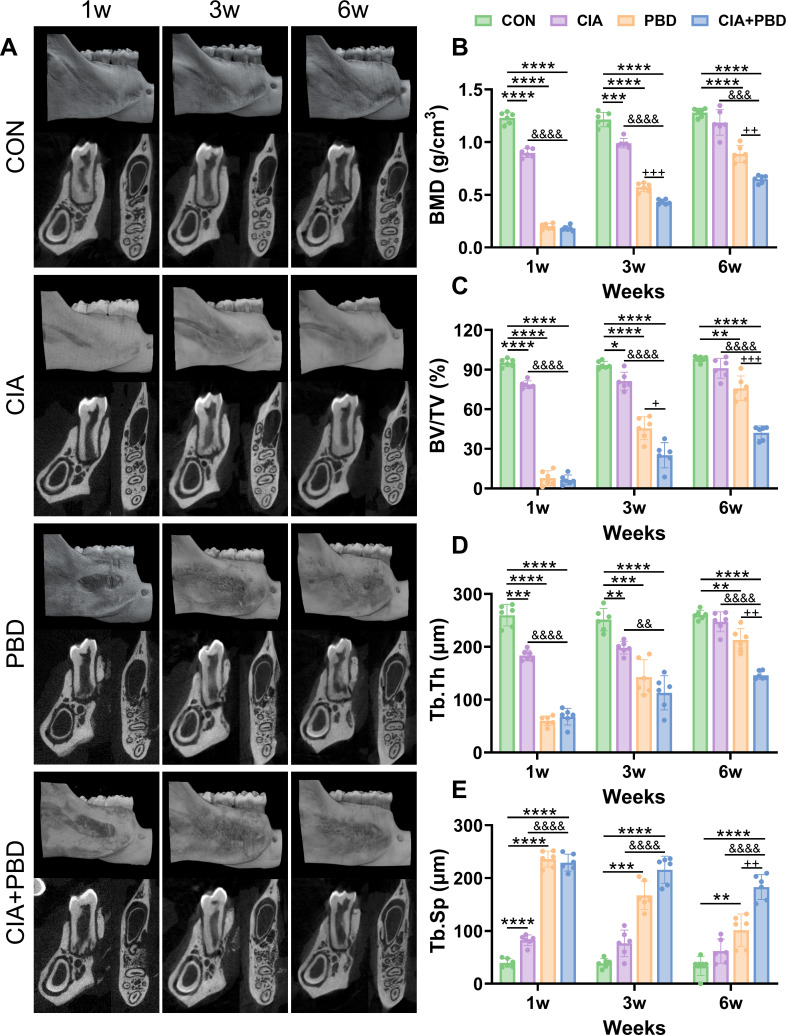
Results of micro-CT (µCT) reconstruction and quantitative analysis of the mandibular defect region. (**A**) µCT reconstructed three-dimensional images of the right mandibular bone defects in coronal planes distal to the first molar and sagittal planes. (**B–E**) Quantitative analysis of bone morphological parameters in periodontal bone defect (PBD) areas.*P<0.05, **P<0.01, ***P<0.001, ****P<0.0001, compared with the control (CON) group; &&P<0.01, &&&P<0.001, &&&&P<0.0001, compared with the collagen-induced arthritis (CIA) group; +P<0.05, ++P<0.01, +++P<0.001, compared with the periodontal bone defect (PBD) group. N=6. BMD, bone mineral density; BV/TV, bone volume/total volume; CIA, collagen-induced arthritis; CON, control; Tb.Sp, trabecular spacing; Tb.Th, trabecular thickness.

Three-dimensional µCT reconstruction of buccolingual sections of the distal mesial roots of the first molar was used for qualitative and quantitative assessment. In three-dimensional µCT reconstructed images, compared with the CON group, the defect areas in PBD-modelled rats displayed varying degrees of bone degeneration, which gradually improved over time. In contrast, the CIA+PBD group demonstrated the poorest healing outcomes in both the coronal and sagittal planes of the mandible at all three time points (figure 1A). Quantitative analysis revealed that (figure 1B–E), in weeks 1 and 3, the CIA group demonstrated significantly lower BMD, BV/TV and Tb.Th than the CON group (p<0.01–0.0001), while Tb.Sp was considerably higher at week 1 (p<0.0001). These findings indicate that the early inflammatory phase of CIA-induced arthritis reduces bone mineralisation and alters trabecular microarchitecture, although gradual healing occurs over time. Compared with the CON group, PBD-modelled rats showed significantly lower BMD, BV/TV and Tb.Th and higher Tb.Sp (p<0.0001–0.001). Over time, BMD, BV/TV and Tb.Th showed recovery, while Tb.Sp gradually decreased. Furthermore, CIA+PBD rats had substantially lower BMD, BV/TV and Tb.Th than PBD rats at weeks 3 and 6 (p<0.001–0.05), while higher Tb.Sp at week 6 (p<0.01), demonstrating that CIA considerably diminishes the osteogenic capacity of alveolar bone. These results confirm the successful establishment of the PBD model and that CIA adversely affects PBD healing.

**Figure 2:**
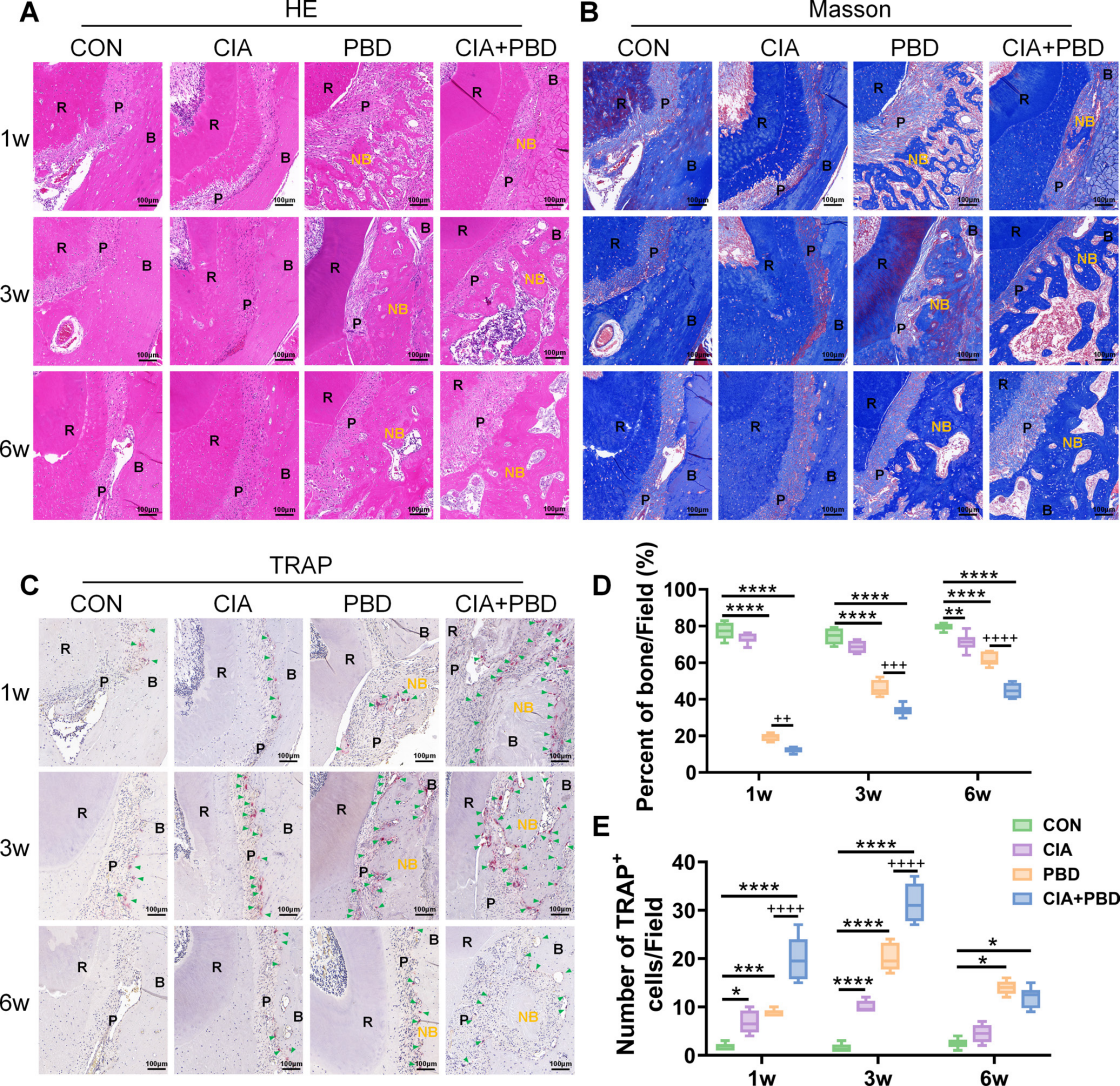
Results of H&E, Masson and tartrate-resistant acid phosphatase (TRAP) staining in mandibular defects. (**A**) H&E and (**B**) Masson staining: new bone formation in the mandibular bone defect at 1, 3 and 6 weeks after surgery. (**C**) TRAP staining: bone resorption in the mandibular bone defect at 1, 3 and 6 weeks after surgery. (**D**) Percentage of trabecular bone area and (**E**) number of osteoclasts in defects. *P<0.05, **p<0.01, ***p<0.001, ****p<0.0001, compared with control (CON) group; ++p<0.01, +++p<0.001, ++++p<0.0001, periodontal bone defect (PBD) versus collagen-induced arthritis (CIA)+PBD group. Scale bar: 100 µm. N=6. B, alveolar bone; green arrows, osteoclasts; NB, newly formed alveolar bone; P, periodontal ligament; R, tooth root.

H&E and Masson staining further revealed progressive formation of striated or irregular new bone trabeculae in the mandibular defect area of PBD rats. Osteoclasts appeared along the margins, accompanied by fibrous and inflammatory tissue surrounding the bone matrix (figure 2A and B). Consistent with µCT findings, new bone formation in the PBD group was superior to that in the CIA+PBD group. Quantification of trabecular bone area revealed significantly reduced values in CIA rats compared with CON rats at week 6 (p<0.01; figure 2D). CIA+PBD rats exhibited consistently and substantially lower bone area than PBD rats at all three time points (week 1: p<0.01; week 3: p<0.001; week 6: p<0.0001). TRAP staining revealed increased osteoclast numbers in all three experimental groups relative to CON rats, with CIA+PBD rats demonstrating the highest osteoclast activity and significantly greater counts than PBD rats at weeks 1 and 3 (weeks 1 and 3: p<0.0001; figure 2C and E).

**Figure 3:**
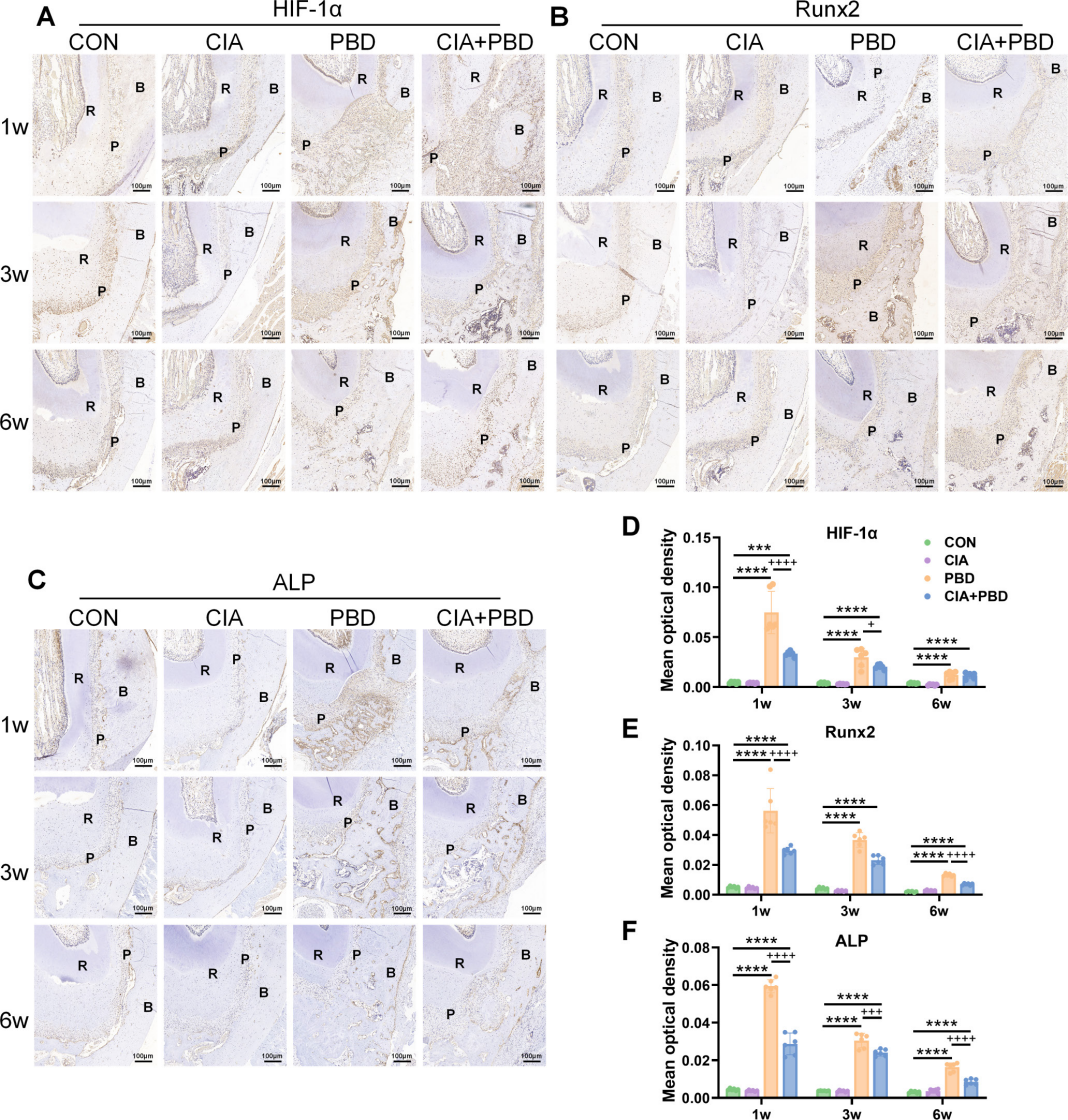
Immunohistochemistry staining results of the mandibular defect area. (**A**) Hypoxia-inducible factor 1 subunit α (HIF-1α), (**B**) runt-related transcription factor 2 (Runx2) and (**C**) alkaline phosphatase (ALP) staining. (**D–F**) Quantitative analysis of average optical density (mean optical density) values. ***p<0.001, ****p<0.0001, compared with control (CON) group; ++p<0.01, ++++p<0.0001, periodontal bone defect (PBD) versus collagen-induced arthritis (CIA)+PBD group. Scale bar: 100 µm. N=6. B, alveolar bone; NB, newly formed alveolar bone; P, periodontal ligament; R, tooth root.

To further assess PBD healing, osteogenesis-related markers were evaluated by IHC. In PBD and CIA+PBD groups, hypoxia-inducible factor 1 subunit α (HIF-1α) and ALP expression increased within the defect region, accompanied by a rise in Runx2-positive cells (figure 3A–C). At all time points, the PBD group exhibited the highest expression levels for all three biomarkers, whereas the CIA+PBD group showed lower levels. Furthermore, expression levels in both groups demonstrated a consistent decline over time (figure 3D–F). Except for ALP in week 3, the above differences at all time points and for all markers reached statistical significance (p<0.05). These findings suggest that the inflammatory microenvironment associated with CIA disrupts the local hypoxic state and impairs HIF-1α expression, thereby inhibiting osteogenic differentiation and bone matrix synthesis.

### RA affects gut microbiota and metabolism in the context of PBD

**Figure 4:**
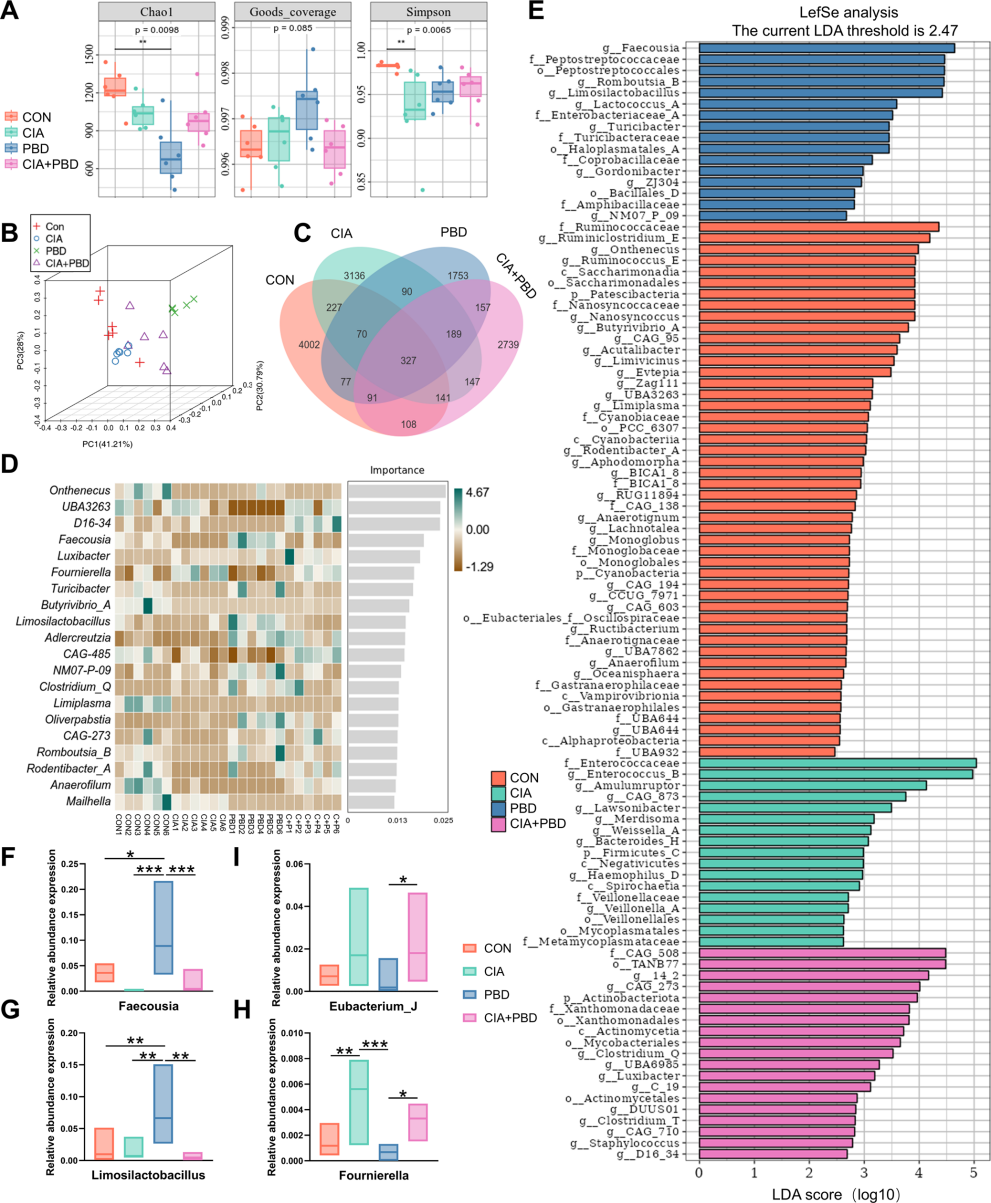
Results of differences in the bacterial flora of rat cecum contents. (**A**) Alpha-diversity analysis: Chao1 index, Goods_coverage index and Simpson index. (**B**) Beta-diversity analysis: principal coordinate analysis (PCoA) analysis of intergroup differences. (**C**) Venn diagram. (**D**) Random forest analysis (scale on the x-axis represents the importance score for genus). (**E**) Linear discriminant analysis effect size (LEfSe) analysis (scale on the x-axis is Linear Discriminant Analysis (LDA) score (log10)). (**F–H**) Relative abundance of *Faecousia*, *Limosilactobacillus*, *Fournierella* and *Eubacterium_J* between four groups. The upper and lower edge lines of each box represent the maximum and minimum values, and the middle line represents the median. *P<0.05, **p<0.01, ***p<0.001. N=6. CIA, collagen-induced arthritis; CON, control; PBD, periodontal bone defect.

To evaluate the impact of CIA on intestinal microbiota, 16S rRNA sequencing was performed on caecal contents from all four groups. Analysis of alpha-diversity revealed that, compared with the CON group, the overall gut microbiota diversity exhibited a decreasing trend across the three experimental groups. Specifically, the Simpson index in the CIA group (p=0.0098) and the Chao1 index in the PBD group (p=0.0065) were both significantly lower than in the CON group, while the Goods_coverage index showed no statistically significant differences among the groups, indicating that gut microbial abundance and diversity were affected to varying degrees following CIA and PBD treatments (figure 4A). Unweighted UniFrac principal coordinate analysis (PCoA) revealed distinct clustering among the four groups, indicating obvious differences in caecal microbiota composition (figure 4B). A total of 327 amplicon sequence variants/OTUs were identified across the four groups (figure 4C). Random forest and LEfSe analyses revealed that *Faecousia* and *Ruminococcus* were substantially enriched in CON rats. However, several genera, including *Fournierella, Enterococcus* and *Bacteroides* were significantly elevated in CIA rats. PBD intervention was associated with increased relative abundance of genera such as *Faecousia, Limosilactobacillus, Romboutsia* and *Lactococcus*, whereas CIA+PBD rats showed enrichment of *Fournierella*, *Eubacterium*_*J*, *Clostridium* and CAG-485 (Linear Discriminant Analysis score >2.4, p<0.05; figure 4D and E). *Faecousia* and *Limosilactobacillus* were reduced in CIA rats but did not differ from CON rats, and were substantially increased in PBD rats, differing significantly from all three other groups (p<0.001–0.05). *Fournierella* and *Eubacterium*_*J* increased in the presence of CIA, with significant differences between PBD and CIA+PBD groups (p<0.05; figure 4F–H). These findings demonstrate substantial differences in gut microbial composition between CIA and healthy rats. CIA-induced intestinal dysbiosis may contribute to the impaired healing of PBDs. These taxa should be interpreted as candidate microbial signatures associated with the observed phenotype, rather than definitive species-level drivers.

**Figure 5:**
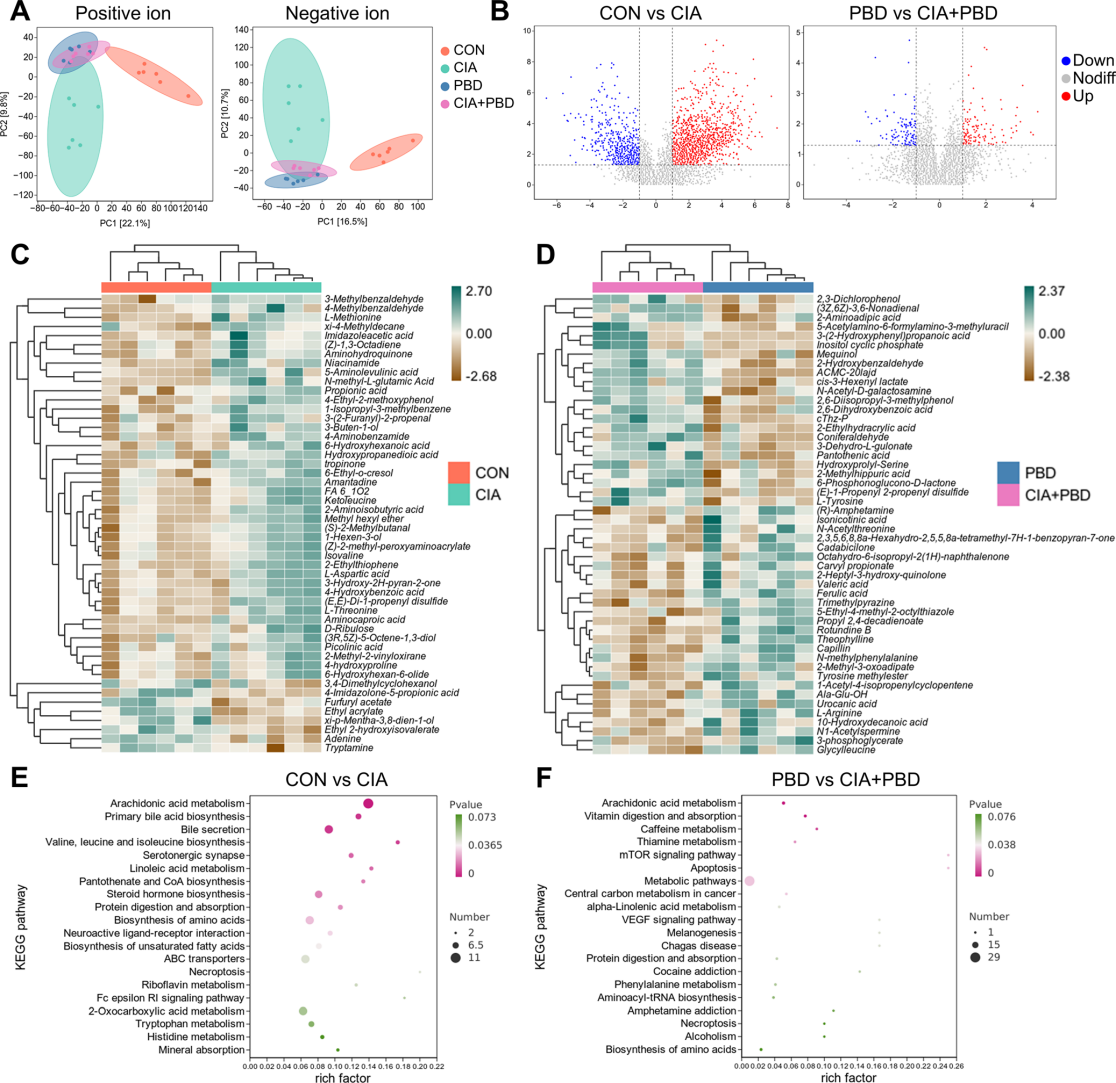
Functional changes in the gut metabolome. (**A**) Orthogonal partial least squares discriminant analysis score plots of metabolic profiles obtained with liquid chromatography-mass spectrometry. (**B**) Volcano plot showing significantly altered metabolites. Heat map showing fold differential metabolite of top 50 variable importance in projection expression: (**C**) control (CON) versus collagen-induced arthritis (CIA) and (**D**) periodontal bone defect (PBD) versus CIA+PBD. (**E, F**) Top 20 of Kyoto Encyclopedia of Genes and Genomes (KEGG) enrichment. N=6. ABC, ATP-binding cassette; CoA, coenzyme; mTOR, mechanistic target of rapamycin; RI, receptor I; tRNA, transfer RNA; VEGF, vascular endothelial growth factor.

To investigate the metabolic effects of CIA and PBD, untargeted metabolomic analysis of caecal contents was performed. OPLS-DA revealed alterations among the four groups in both positive and negative modes (positive mode: R²Y=0.992, Q²=0.892; negative mode: R²Y=0.992, Q²=0.882). CIA and CON groups displayed the most distinct metabolic profiles. Conversely, PBD and CIA+PBD groups partially overlapped but remained distinguishable from CIA and CON groups (figure 5A). Volcano plots suggested that, in the CON versus CIA comparison, 343 metabolites were upregulated, including 2-aminoadipic acid, 10Z-heptadecenoic acid, pregnenolone sulfate, aldosterone, corticosterone, tryptophan, 5-hydroxy-L-tryptophan (5-HT) and indole, and 182 were downregulated, such as prostaglandin G_2_ (PGG_2_), dehydroepiandrosterone sulfate and tryptamine. In the PBD versus CIA+PBD comparison, 62 metabolites were upregulated and 52 downregulated, with increases in 2-aminoadipic acid, 10Z-heptadecenoic acid, adrenosterone and 5-HT, and decreases in prostacyclin (PGI_2_), 5-keto-eicosatetraenoic acid and androstanedione (figure 5B). Using variable importance in projection >1 and p<0.05 thresholds, 339 and 65 differential metabolites were identified in the CON versus CIA and PBD versus CIA+PBD comparisons, respectively. The top 50 metabolites (figure 5C and D) showed upregulation of L-methionine, L-tryptophan, L-aspartic acid, niacinamide, 5-aminolevulinic acid and hydroxypropanoic acid, and downregulation of adenine in CIA rats (figure 5C). In CIA+PBD rats, L-arginine, N1-acetylspermidine, ferulic acid and 3-phosphoglycerate were increased, while pantothenic acid was reduced (figure 5D). It is noteworthy that, although there are shared changes in metabolite directionality, the RA-associated metabolic alterations observed in the context of localised PBD injury are more subtle compared with the pronounced metabolic dissociation seen in systemic CIA models. The three most enriched pathways in CON versus CIA comparison were arachidonic acid metabolism, primary bile acid biosynthesis and bile secretion (figure 5E). Within arachidonic acid metabolism, arachidonic acid, dehydroketone prostaglandin E_2_ 20-hydroxy-leukotriene B_4_, lipoxin B_4_ (LXB_4_), 2,3-Dinor-8-iso-prostaglandin F2α, 15(S)-hydroxyeicosatetraenoic acid (15(S)-HETE) and leukotriene E_4_ were upregulated, whereas PGG_2_ and 20-HETE were downregulated. For the PBD versus CIA+PBD comparison, the most enriched pathways were arachidonic acid metabolism, vitamin digestion and absorption and caffeine metabolism, with additional enrichment of the mechanistic target of rapamycin (mTOR) pathway (figure 5F). In this context, prostaglandin J_2_, leukotriene B_4_ (LTB_4_) and LXB_4_ were upregulated, while PGI_2_ and prostaglandin A_2_ were downregulated. These results suggest that CIA alters gut microbiota-associated metabolism, particularly arachidonic acid pathways, which may impair healing of PBDs.

### Successful construction of the pseudo-germ-free rat model and FMT system

A marked depletion of gut commensal bacteria in antibiotic-treated rats was confirmed by 16S rRNA gene sequencing. The alpha-diversity was substantially reduced in the PGF-1w group compared with the CON-1w group (p<0.01; online supplemental figure S3A). Relative abundance analysis revealed that most pre-existing bacterial communities were eliminated (online supplemental figure S3B). Hierarchical clustering further suggested that PGF-1w samples formed a distinct cluster, clearly separated from CON-1w samples (online supplemental figure S3C). These findings collectively confirmed effective depletion of gut symbiotic bacteria and successful establishment of a PGF state in antibiotic-treated rats.

**Figure 6:**
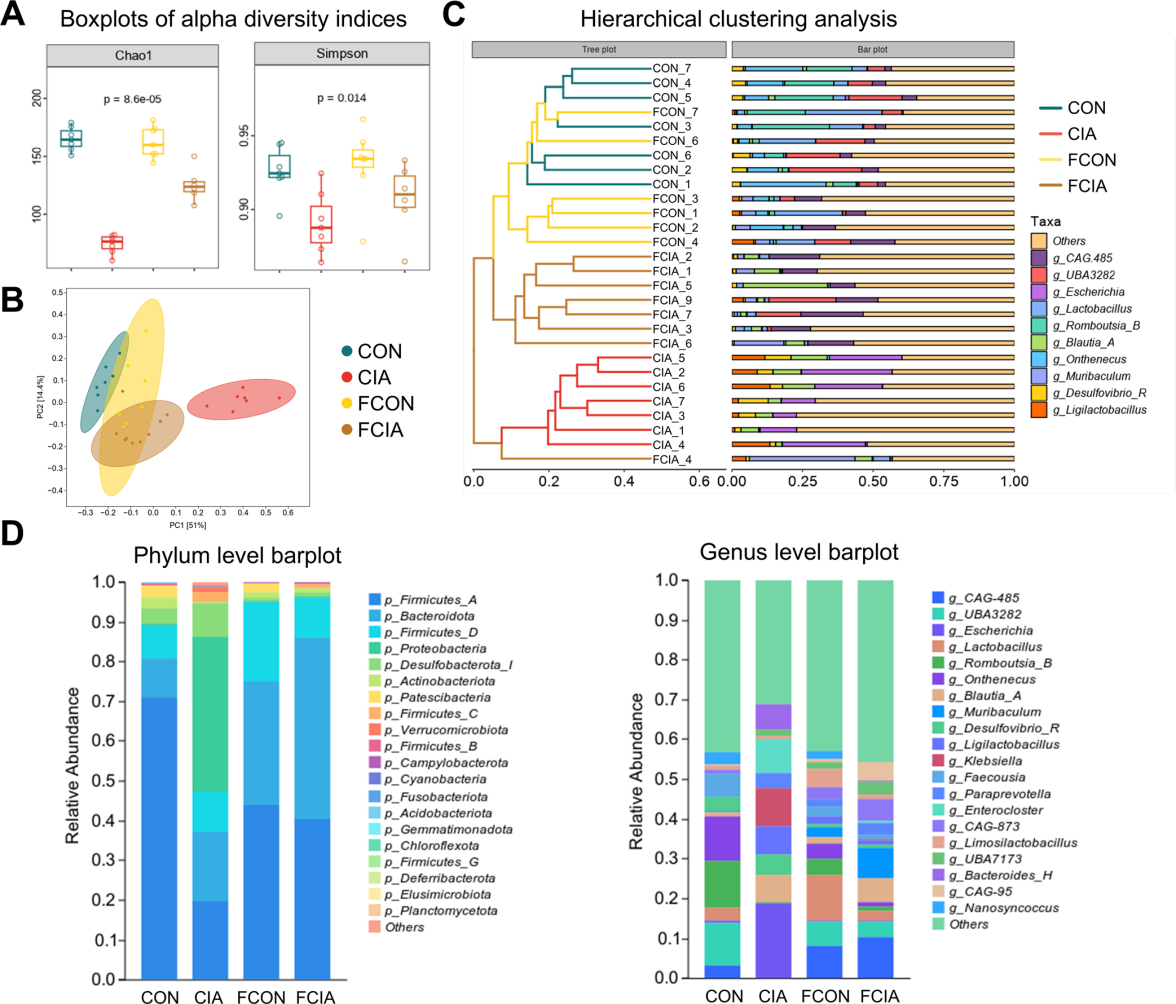
16S ribosomal RNA (rRNA) sequencing results of cecal contents from rats after faecal microbiota transplantation (FMT). (**A**) Alpha-diversity analysis: Chao1 index and Simpson index. (**B**) Unweighted UniFrac principal coordinate analysis (PCoA). (**C**) Hierarchical cluster analysis based on unweighted Unifrac distance. (**D**) Relative abundance analysis at the phylum level and genus level. N=6. CIA, collagen-induced arthritis; CON, control; FCIA, FMT from CIA donors; FCON, FMT from healthy control donors.

Gut microbiota from donor rats were transplanted into antibiotic-treated recipients by oral gavage. Following FMT, the diversity indexes of the FCON and FCIA groups approached those of their respective donor groups (figure 6A). Clustering tree and PCoA analyses showed that FCON samples clustered together with the CON group, whereas FCIA samples clustered with CIA rats, suggesting that the microbial communities of FMT recipients resembled those of their donors (figure 6B and C). FCON and CON groups displayed comparable distributions of dominant bacterial genera; FCIA and CIA groups exhibited similar dysbiosis patterns, including changes in the Firmicutes/Bacteroidota ratio and increases in several opportunistic pathogens (figure 6D).

### FMT from CIA donor rats inhibits the healing of PBD

For faecal transplantation experiments, a donor CIA model was established following previously described methods. As shown in online supplemental figure S4A, rats in the CIA group exhibited marked ankle-joint damage, including structural destruction and indistinct joint margins due to severe bone erosion. No substantial joint abnormalities were observed in the remaining three groups. Consistent with these findings, CIA rats demonstrated significantly reduced BMD, BV/TV and Tb.Th, and substantially increased Tb.Sp compared with all comparisons (p<0.0001; online supplemental figure S4B-E). Pathological staining (online supplemental figure S5A) revealed narrowing of the ankle joint space, synovial membrane thickening, cartilage surface disruption and pannus formation in CIA rats. Subchondral bone erosion, loss of trabecular continuity and significant osteoclast accumulation were observed. Quantitative analyses remained consistent with these findings (online supplemental figure S5B-E), confirming the successful establishment of the CIA model in donor rats used for faecal transplantation.

**Figure 7:**
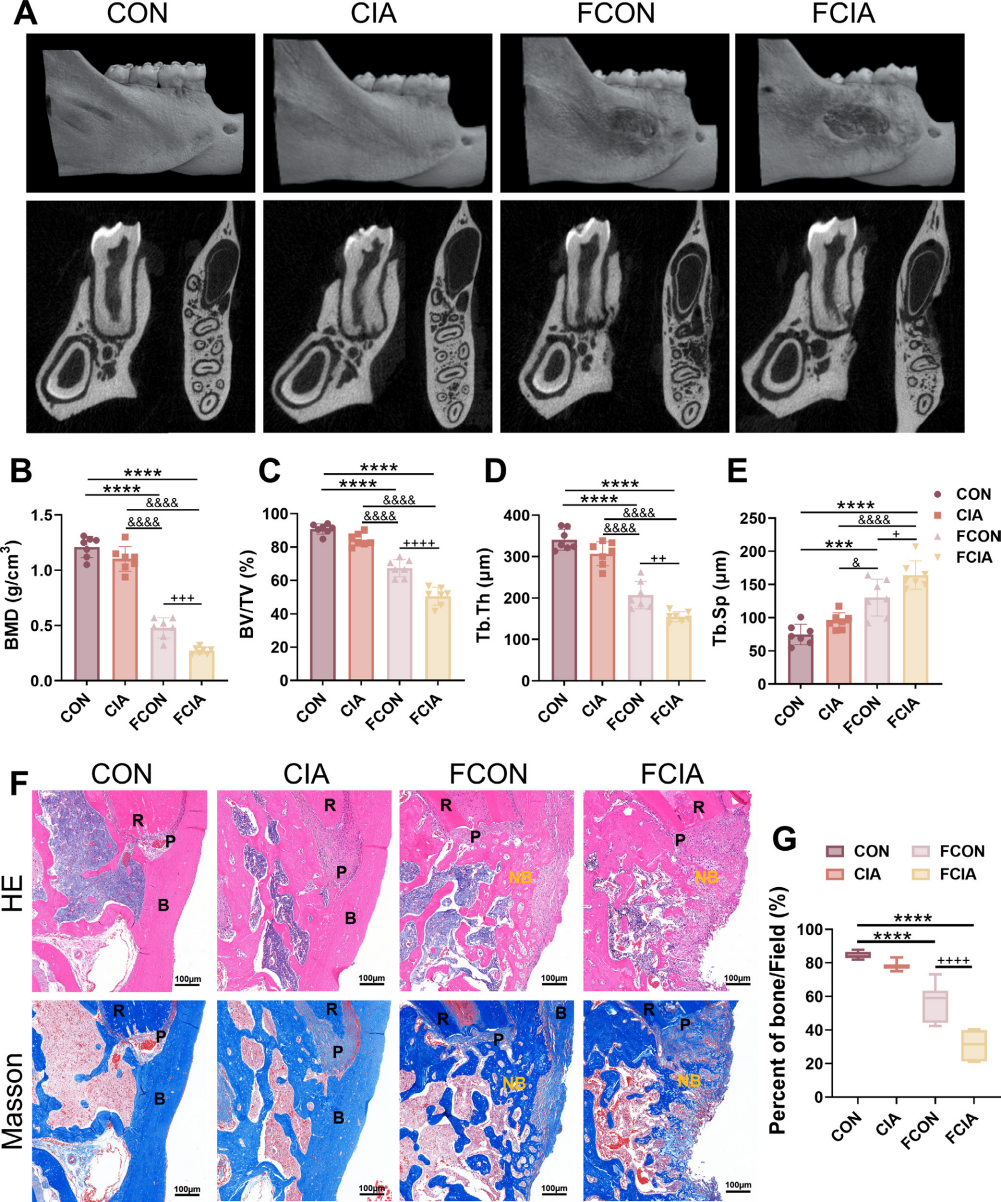
Faecal microbiota transplantation (FMT) from collagen-induced arthritis (CIA) donor rats inhibits the healing of periodontal bone defect (PBD). (**A**) Micro-CT (µCT) reconstruction of the mandibular defect area. (**B–E**) Quantitative analysis of relevant bone parameters . (**F**) Pathological images of mandibular bone stained with H&E and Masson (scale bar: 100 µm; B, alveolar bone; NB, newly formed alveolar bone; P, periodontal ligament; R, tooth root). (**G**) Percentage of bone trabecular area in the region of mandibular bone defects. ***P<0.001, ****P<0.0001, compared with the control (CON) group; &P<0.05, &&&&P<0.0001, compared with the collagen-induced arthritis (CIA) group; +P<0.05, ++P<0.01, +++P<0.001, ++++P<0.0001, compared with the FMT from healthy control donors (FCON) group. FCIA, FMT from CIA donors. N=7. BMD, bone mineral density; Tb.Sp, trabecular spacing; Tb.Th, trabecular thickness.

**Figure 8:**
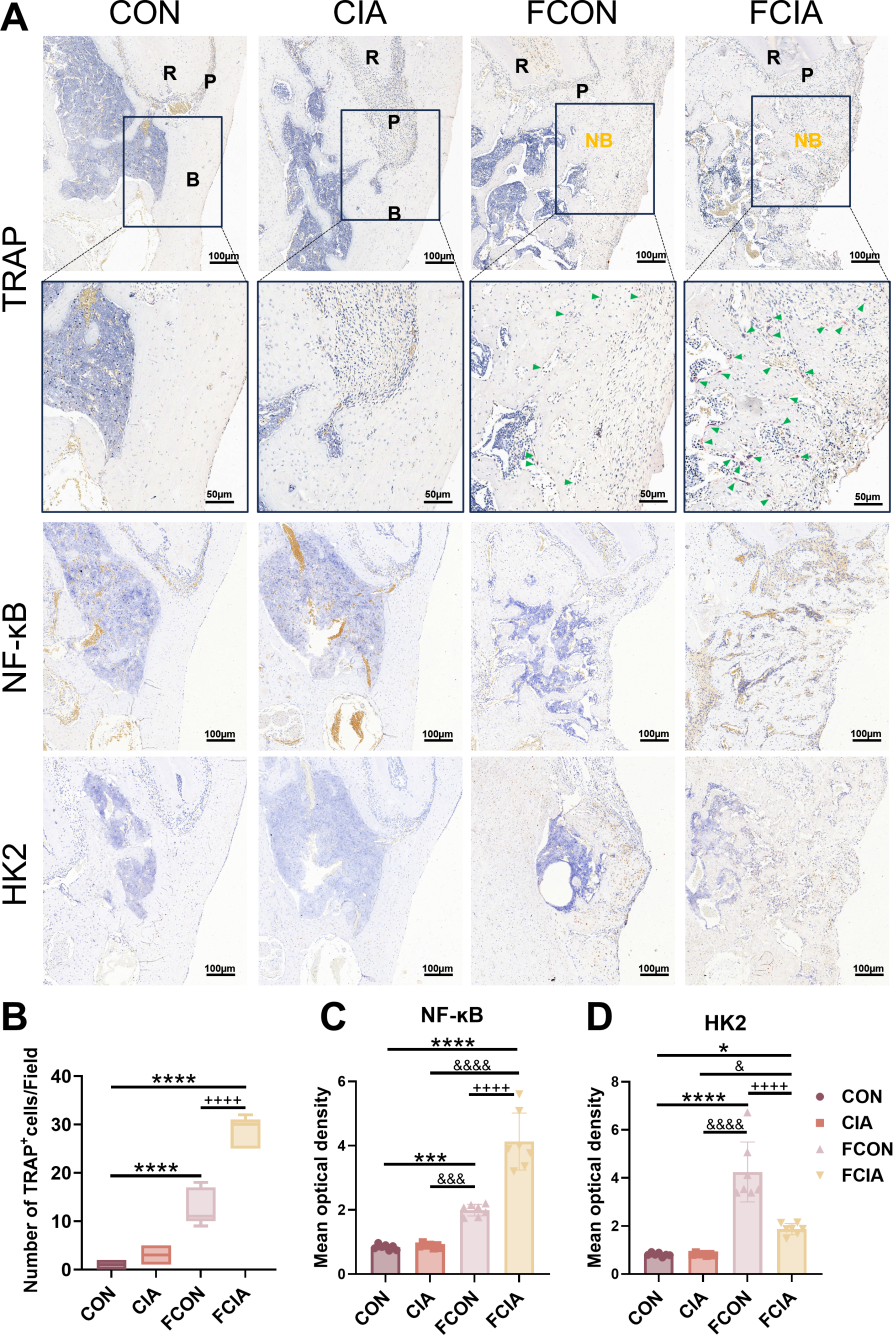
Tartrate-resistant acid phosphatase (TRAP) and immunohistochemistry staining results of the mandibular defect area in rats. (**A**) TRAP, nuclear factor kappa-B (NF-ĸB) and hexokinase 2 (HK2) staining (scale bar: 50 and 100 µm; B, alveolar bone; green arrows, osteoclasts; NB, newly formed alveolar bone; P, periodontal ligament; R, tooth root). (**B**) Number of osteoclasts in the mandibular bone defect area. (**C, D**) Quantitative analysis of mean optical density values. *P<0.05, ***P<0.001, ****P<0.0001, compared with the control (CON) group; &P<0.05, &&&P<0.001, &&&&P<0.0001, compared with the collagen-induced arthritis (CIA) group; ++++P<0.0001, compared with the FMT from healthy control donors (FCON) group. FCIA, FMT from CIA donors. N=7.

Preliminary experimental results indicated that CIA impacted PBD healing. To investigate whether this effect is mediated by gut microbiota, caecal contents from CON and CIA rats were orally gavaged into PGF-PBD rats, and mandibular defect healing was assessed. The µCT reconstruction showed that FCIA rats exhibited reduced new bone formation compared with FCON rats (figure 7A). FCIA rats showed significantly lower BMD, BV/TV and Tb.Th, and higher Tb.Sp than FCON rats (p<0.0001–0.05; figure 7B–E). H&E and Masson staining revealed a significantly smaller proportion of trabecular bone area in FCIA rats (p<0.0001; figure 7F and G). TRAP staining further showed that osteoclast numbers were substantially higher in FCIA rats than in the other groups (p<0.0001; figure 8A and B). IHC suggested increased expression of NF-ĸB and HK2 in both FCON and FCIA groups, with significant differences between them (p<0.0001). The NF-ĸB levels were highest in FCIA rats, whereas HK2 expression was highest in FCON rats (figure 8C and D). Collectively, these findings indicate that FMT from CIA donors inhibit healing of PBDs, suggesting that CIA impairs bone regeneration, at least in part, by altering gut microbial composition. To further investigate the functional consequences of gut microbiota alterations, we performed an in vitro stimulation assay using supernatants derived from intestinal contents (online supplemental figure S6). LPS (1 μg/mL) showed a marked reduction in cell viability. For FMS from CON animals, FMS-100 significantly reduced cell viability at all the time points. In contrast, FMS from CIA animals showed a consistent association with decreased cell viability across tested concentrations and time points, indicating a stronger inhibitory effect on macrophage viability under RA-associated conditions. Based on the results of CCK-8, FMS-100 from CON and CIA rats were selected for the following experiment. Macrophages exposed to CIA FMS exhibited increased expression of pro-inflammatory cytokines, including TNFα and IL-1β, compared with controls (online supplemental figure S7). These findings suggest that gut microbiota-associated soluble factors under RA conditions can promote macrophage activation and inflammatory responses. This functional observation complements the FMT results and supports a link between gut dysbiosis and systemic immune activation.

To further explore the relationship between microbial alterations, metabolic changes and bone microarchitecture, we performed Spearman’s correlation analysis (online supplemental figure S8). Bone structural parameters, including BMD and BV/TV, showed significant negative correlations with pro-inflammatory microbial taxa and metabolites, such as *Fournierella* and LTB_4_, and positive correlations with potentially beneficial taxa such as *Limosilactobacillus*. Conversely, Tb.Sp, a marker of bone deterioration, was positively correlated with *Fournierella* and LTB_4_, and negatively associated with *Limosilactobacillus*. Notably, *Fournierella* exhibited a strong positive correlation with LTB_4_, suggesting a potential link between dysbiotic microbial signatures and pro-inflammatory lipid mediators. Taken together, these findings indicate that RA-associated microbial dysbiosis and metabolic alterations are closely associated with changes in bone microarchitecture.

## Discussion

Recent studies have highlighted a bidirectional association between periodontitis and RA. However, the specific effect of RA on periodontal regeneration, an important but challenging aspect of periodontitis management, remains insufficiently defined. In this study, we evaluated PBD healing in a CIA rat model and explored the potential involvement of gut microbiota. Our findings suggest that (1) CIA is associated with impaired periodontal bone formation, particularly at early stage; (2) the PBD model does not exacerbate CIA, likely reflecting its acute, non-inflammatory nature and (3) transplantation of microbiota from CIA donors is associated with reduced regenerative capacity. Collectively, these results support a potential link between RA-associated dysbiosis and impaired periodontal healing.

Gut microbial dysbiosis has been consistently reported in RA and is closely associated with immune dysfunction. Previous studies have identified enrichment of microbial taxa such as *Collinsella*, *Enterococcus* and *Sedimentibacter* in RA,^30 31^ while other reports have described alterations in genera including *Eubacterium* and *Bacteroides*.^32^ The functional roles of these taxa appear to be context-dependent.^33 34^ In our study, similar patterns of microbial alteration were observed at the genus level. However, given the resolution limits of 16S rRNA sequencing, these taxa should be interpreted as candidate microbial signatures rather than definitive species-level drivers. Metabolomic analysis further revealed alterations in several pathways, including arachidonic acid metabolism, mTOR signalling and vitamin-related pathways. Increased levels of lipid mediators such as LTB_4_ and PGE_2_ are consistent with a pro-inflammatory metabolic environment, while reduced short-chain fatty acid-associated signals suggest disruption of anti-inflammatory microbial metabolism.^35^,^38^ Together, these findings indicate a shift in systemic metabolic homeostasis under RA conditions.

Our FMT experiments further support the contributory role of gut microbiota in periodontal repair. Transplantation of microbiota from CIA donors into antibiotic-treated recipients was associated with impaired bone healing, reduced osteogenic marker expression and increased osteoclast activity. However, these findings should be interpreted with caution, as the experimental design does not fully exclude potential confounding effects, including antibiotic pretreatment and host systemic influence.

To further explore the functional implications of these observations, we performed in vitro stimulation experiments using FMS. Macrophages exposed to CIA-FMS showed increased expression of TNFα and IL-1β, suggesting that gut-derived factors are associated with enhanced pro-inflammatory responses. These findings complement the FMT results and provide functional support for a link between gut microbiota alterations and systemic immune activation. These observations are consistent with our correlation analysis, in which pro-inflammatory taxa and metabolites, such as *Fournierella* and LTB_4_, were associated with adverse bone structural parameters, whereas potentially beneficial taxa such as *Limosilactobacillus* showed positive associations. However, these findings are based on correlation analysis and do not establish causality. Rather, our results provide system-level framework functional support for a link between gut microbiota alterations and systemic immune activation. From a mechanistic perspective, TNFα and IL-1β are key regulators of inflammatory signalling and have been reported to be associated with metabolic pathways identified in this study. These cytokines may enhance arachidonic acid metabolism by promoting the production of lipid mediators such as LTB_4_ and PGE_2_, thereby contributing to inflammatory cascades and osteoclastogenesis.^39^,^42^ In addition, TNFα and IL-1β may interact with mTOR signalling pathways to influence immune cell metabolism and differentiation.^43^,^45^

The intestinal microbiota contributes to the synthesis and metabolism of several vitamins, such as vitamin K_2_ and multiple B vitamins, which influence bone mass and bone matrix quality.^46^ It is noteworthy that although elevated levels of certain vitamin B-related metabolites were observed in the caecal contents, this does not necessarily indicate their availability or activity at the site of periodontal defects. The systemic effects of RA, including chronic inflammation and vascular dysfunction, may limit the transport or utilisation of these metabolites within local tissues, thereby potentially restricting their contribution to bone regeneration.

Furthermore, increased NF-ĸB and HK2 expressions in the mandibular defect regions suggest that microbiota from CIA donors linked to activated NF-ĸB-driven inflammation and glycolytic pathways locally. Consistent with this, reduced HIF-1α expression observed in the CIA+PBD group may reflect impaired adaptive hypoxic signalling rather than reduced inflammation. Although inflammation is often associated with HIF-1α induction, chronic systemic stress may impair its pro-regenerative function.^22^ Reduced expression of Runx2 and ALP, coupled with a substantial rise in osteoclast numbers, indicates suppression of osteogenic gene activity and enhanced bone resorption. Collectively, these findings suggest that gut microbial dysbiosis is closely associated with compromised periodontal regeneration and highlight microbiota modulation as a potential therapeutic strategy with important clinical implications. Concurrently, pharmacological modulation of key pathways, such as arachidonic acid metabolism and mTOR signalling, may provide additional therapeutic benefit. Ultimately, combining systemic pharmacological control with microbiome-targeted therapies may establish a multidimensional ‘*immuno-metabolism-microecology*’ treatment framework. Notably, our findings can be viewed within a maladaptive trained immunity framework. While periodontitis has been shown to ‘train’ haematopoietic progenitors to enhance arthritis,^47 48^ our data suggest a reciprocal process whereby RA-associated dysbiosis primes the periodontal microenvironment towards a persistent pro-inflammatory, pro-resorptive state, impairing healing. However, as no direct assessment of immune reprogramming was performed, this interpretation remains conceptual and requires further validation.

Although the present study primarily focused on the gut-to-bone direction, the potential contribution of the oral-gut axis should also be considered. Previous studies have demonstrated that oral pathogens such as *P. gingivalis* can modulate gut microbiota composition and exacerbate arthritis.^49^ In our previous study, we transplanted periodontitis-associated salivary microbiota and observed aggravated RA via an oral-gut-immune axis.^48^ In the present study, however, gut microbiota was directly manipulated through antibiotic pretreatment followed by FMT via oral gavage, allowing targeted modulation of intestinal microbial communities. The resulting phenotypic changes therefore support a direct contribution of gut dysbiosis to impaired periodontal healing. Thus, our findings should be interpreted as demonstrating that gut dysbiosis is sufficient to impair periodontal regeneration, without establishing it as the exclusive driver.

Several limitations should be acknowledged. First, the PBD model represents an acute, surgically induced injury rather than chronic biofilm-driven periodontitis and therefore does not fully recapitulate clinical disease.^50^ Instead, it provides a controlled framework to evaluate how systemic conditions influence regenerative capacity.^51^ Second, microbiome analysis was based on 16S rRNA sequencing, which provides genus-level resolution and was not independently validated by targeted approaches. Third, although FMT experiments support a role for gut microbiota, they do not establish causality. Finally, given the relatively modest sample size for multi-omics analyses, these findings should be considered exploratory. Future studies incorporating larger cohorts, metagenomic sequencing, targeted metabolomics and mechanistic validation will be required to confirm these observations.

In conclusion, our findings suggest that RA-associated gut dysbiosis and metabolic alterations are associated with impaired periodontal bone regeneration. These results provide a framework for understanding how systemic autoimmune conditions may influence tissue repair and highlight the importance of considering host systemic status in periodontal therapy.

## Supplementary material

10.1136/rmdopen-2026-006931 online supplemental file 1

## Data Availability

Data are available in a public, open access repository. Data are available on reasonable request.
