# Supplementary material for: Gut microbiota dysbiosis and metabolic alterations in rheumatoid arthritis: a barrier to periodontal repair
Source: RMD Open. 2026 May 21;12(2):e006931. doi: 10.1136/rmdopen-2026-006931 (PMC13202092; doi:10.1136/rmdopen-2026-006931)
Supplement: online supplemental file 1 [file rmdopen-12-2-s001.pdf]

Gut Microbiota Dysbiosis and Metabolic Alterations in Rheumatoid Arthritis: A Barrier to Periodontal Repair

Di Cui[1,2#,*], Yingying Zhou[1#], Yibing Zhou[1], Ruiyang Ge[1], Haowei Mao[1], Motilal Mathesh[2], Lei Han[1*], Wenrong Yang[2], Fuhua Yan[1*]

[1]Nanjing Stomatological Hospital, Affiliated Hospital of Medical School, Institute of Stomatology, Nanjing University, Nanjing, 210008, China

[2]School of Life and Environmental Sciences, Centre for Sustainable Bioproducts, Deakin University Waurn Ponds, Victoria, 3216, Australia

Di Cui[#] and Yingying Zhou[#] contributed equally to this work and should be regarded as co-first authors.

*** Correspondence:**
Fuhua Yan[*] yanfh@nju.edu.cn
Lei Han[*] pinedream@163.com
Di Cui* d.cui@deakin.edu.au

Supplementary Material

Supplementary Figures

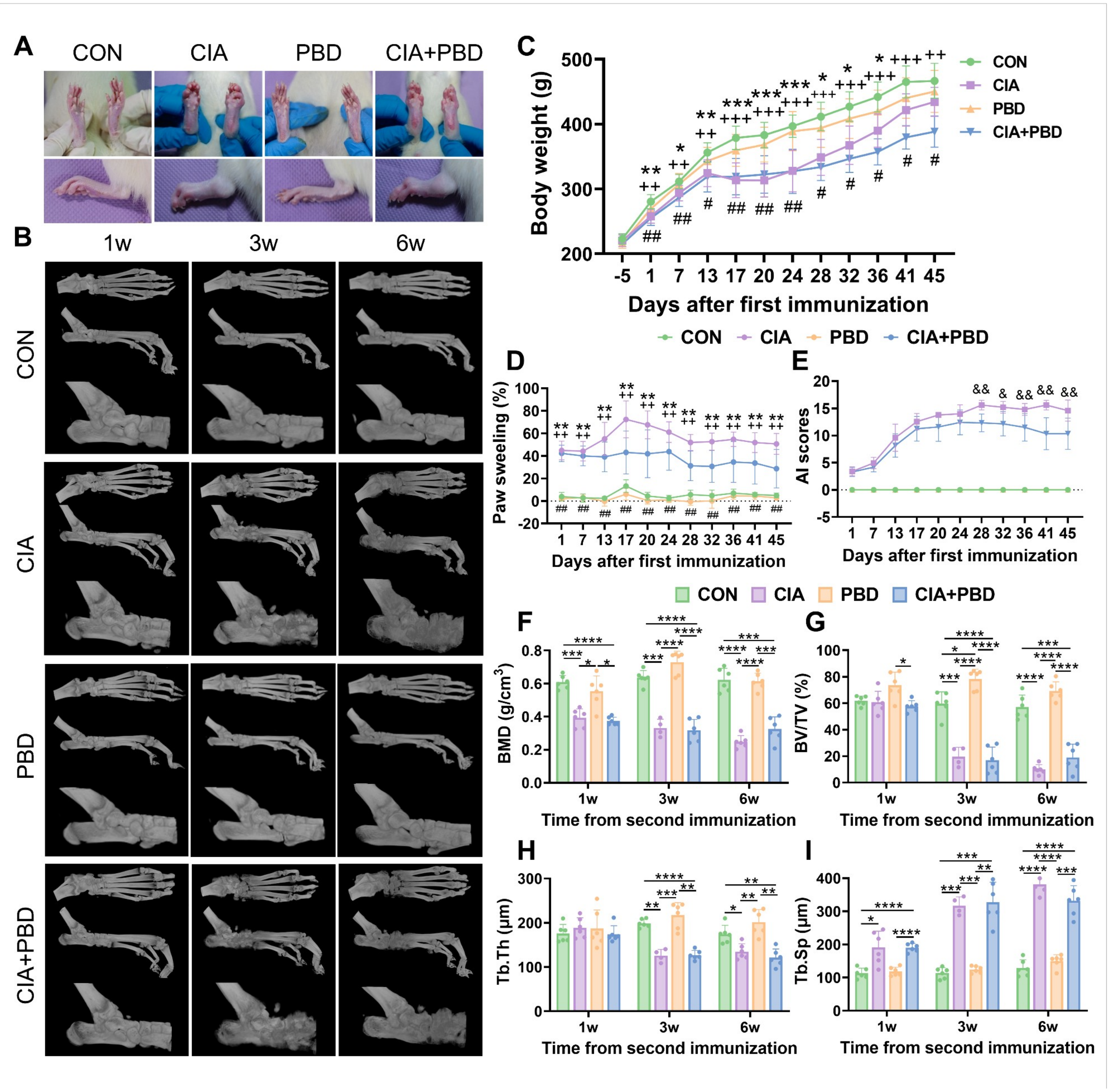

Supplementary Figure S1. Clinical assessment of joint inflammation and μCT findings. (A) Comparison of typical macroscopic paw images of rats. (B) Reconstructed 3D μCT images of ankle joints at 1, 3 and 6 weeks after surgery. Data of (C) body weight, (D) paw swelling and (E) AI scores of rats in each group at different time points after the first immunization (**P*<0.05, ***P*<0.01, ****P*<0.001, *****P*<0.0001, CIA group vs. CON group; +*P*<0.05, ++*P*<0.01, +++*P*<0.001, ++++ *P*<0.0001, CIA+PBD group vs. CON group; #*P*<0.05, ##*P*<0.01, ###*P*<0.001, ####*P*<0.0001,

PBD group vs. CIA+PBD group; &P<0.05, &&P<0.01, CIA group vs. CIA+PBD group). (F-I) Quantitative analysis of bone morphology indexes of the ankle joint (*P<0.05, **P<0.01, ***P<0.001, ****P<0.0001). n= 6.

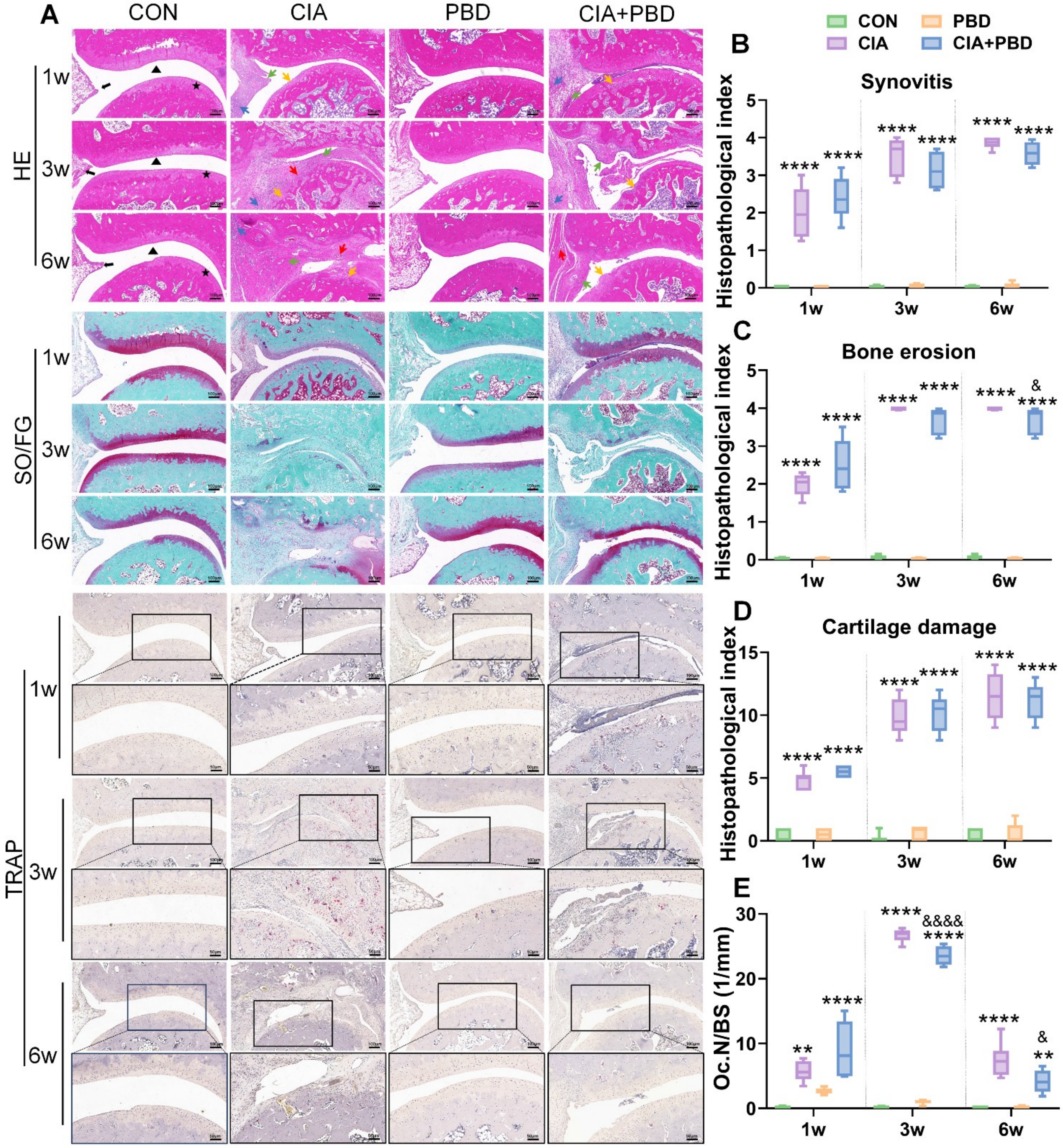

Supplementary Figure S2. HE, SO-FG and TRAP staining results. (A) Typical HE, SO-FG and TRAP staining photos of ankle joint sections (Scale bars: 50 and 100 μm. The image below is an enlarged version of the image in the black box above. Black arrow: synovial tissue; Triangle: joint cavity; Pentagram: hyaline cartilage; Red arrow: pannus; Yellow arrow: cartilage tissue damage; Blue arrow: inflammatory cell infiltration; Green arrow: synovial tissue hyperplasia). (B, C) Pathological change evaluation results: synovitis and bone erosion. (D) Mankin score results of cartilage damage. (E)

Number of osteoclasts per bone surface (Oc.N: number of osteoclasts; BS: bone surface). ***P*<0.01, *****P*<0.0001, compared with CON group. &*P*<0.05, &&&&*P*<0.0001, CIA vs. CIA+PBD group. n= 6.

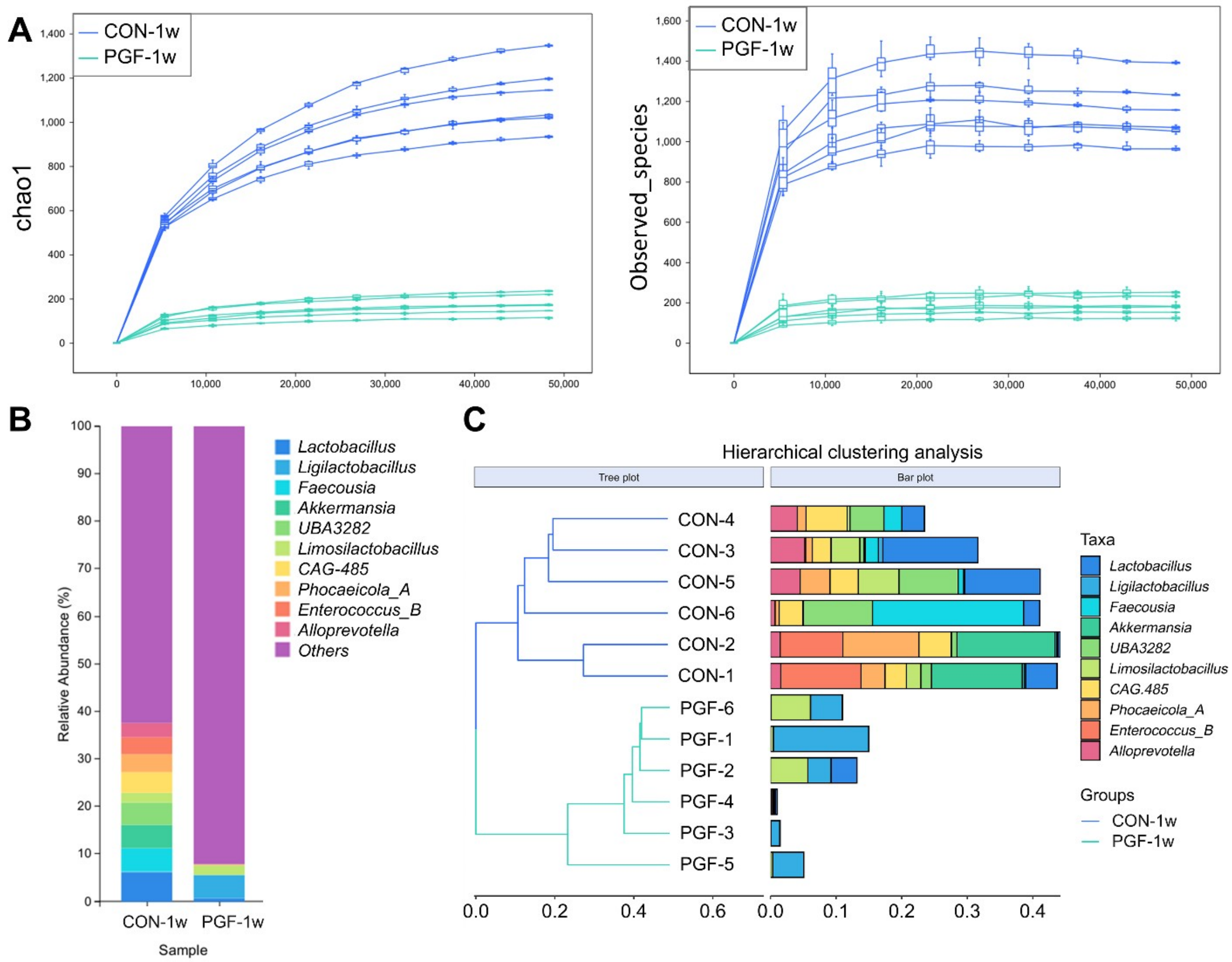

Supplementary Figure S3. 16S rRNA sequencing results of caecal contents from antibiotic-treated rats. (A) α-diversity analysis. (B) Relative abundance analysis at genus level. (C) Hierarchical cluster analysis based on unweighted Unifrac distance. n=6.

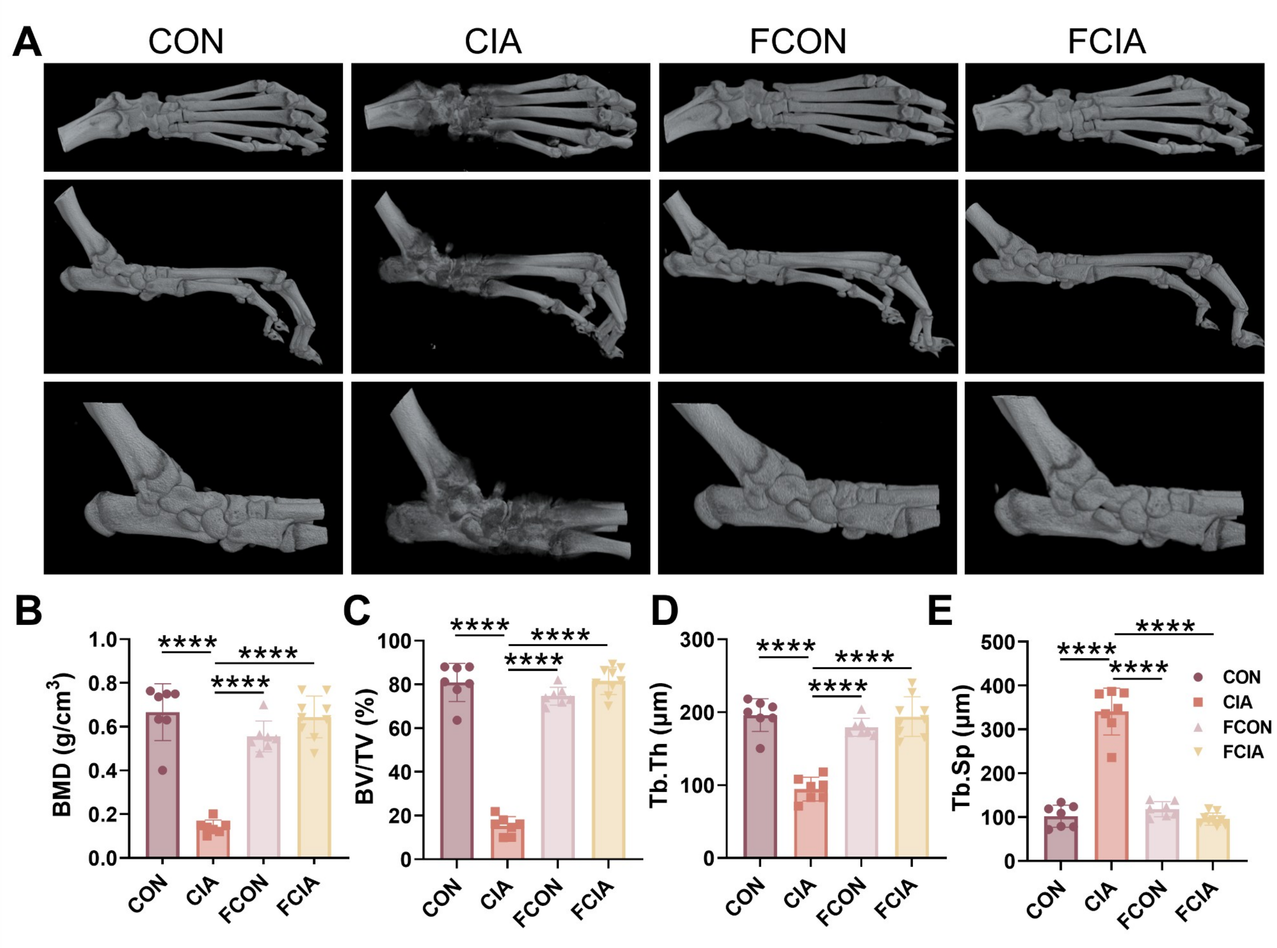

Supplementary Figure S4. Validation of the CIA model in FMT donor rats. (A) μCT reconstruction of the ankle joint. (B-E) Quantitative analysis of bone parameters of the ankle joint (****$P<0.0001$). n=7.

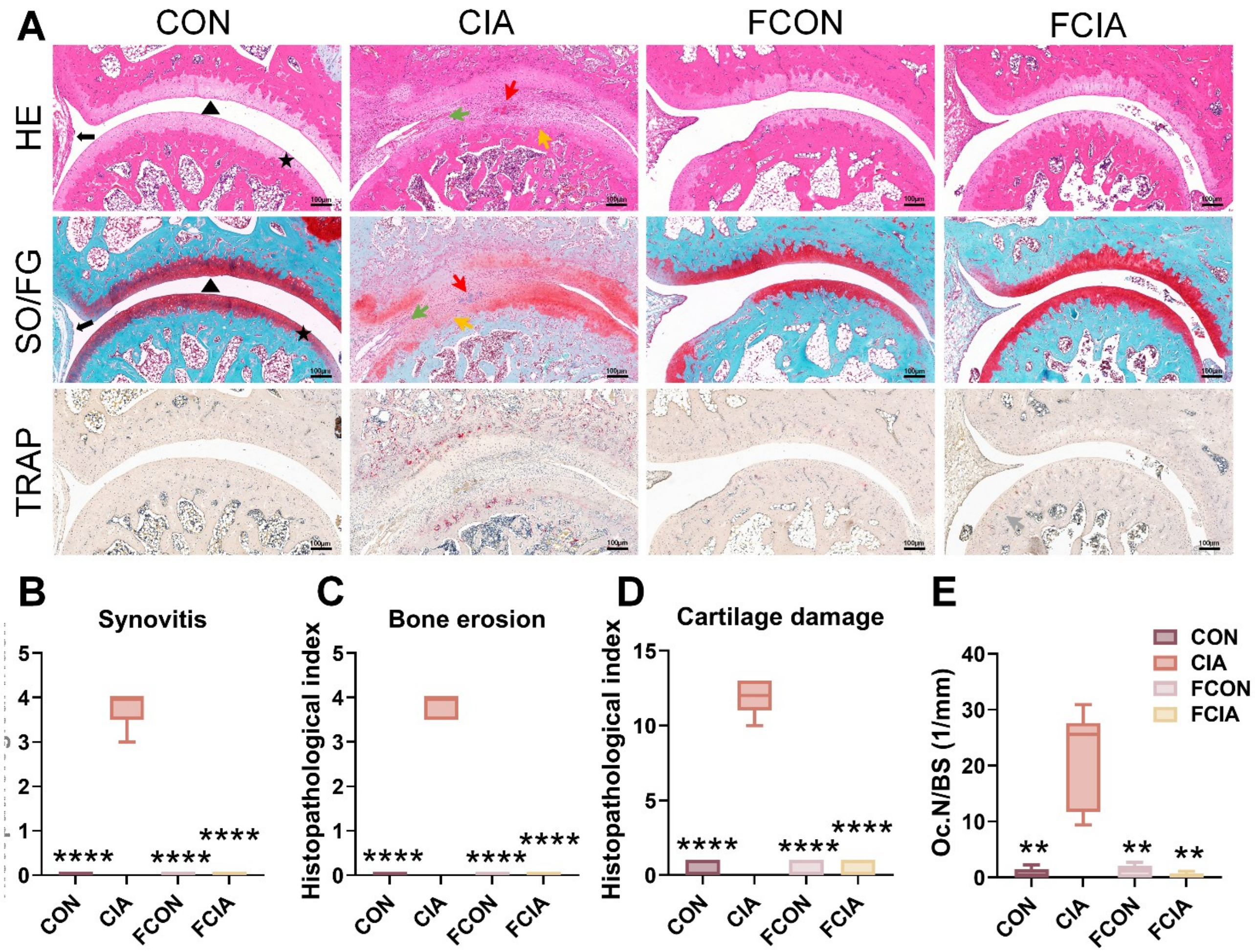

Supplementary Figure S5. Histopathological staining results of ankle joint in faecal microbiota transplant donor rats. (A) Pathological images of HE, SO-FG and TRAP staining of the ankle joint (Scale bars: 100 μm. Black arrow: synovial tissue; Triangle: joint cavity; Pentagram: hyaline cartilage; Red arrow: pannus; Yellow arrow: cartilage tissue damage; Green arrow: synovial tissue hyperplasia). (B, C) Assessment of joint inflammation: synovitis and bone erosion. (D) Mankin score of cartilage damage. (E) Number of osteoclasts per bone surface (Oc.N: number of osteoclasts; BS: bone surface). ***P*<0.01, *****P*<0.0001, compared with CIA group. n=7.

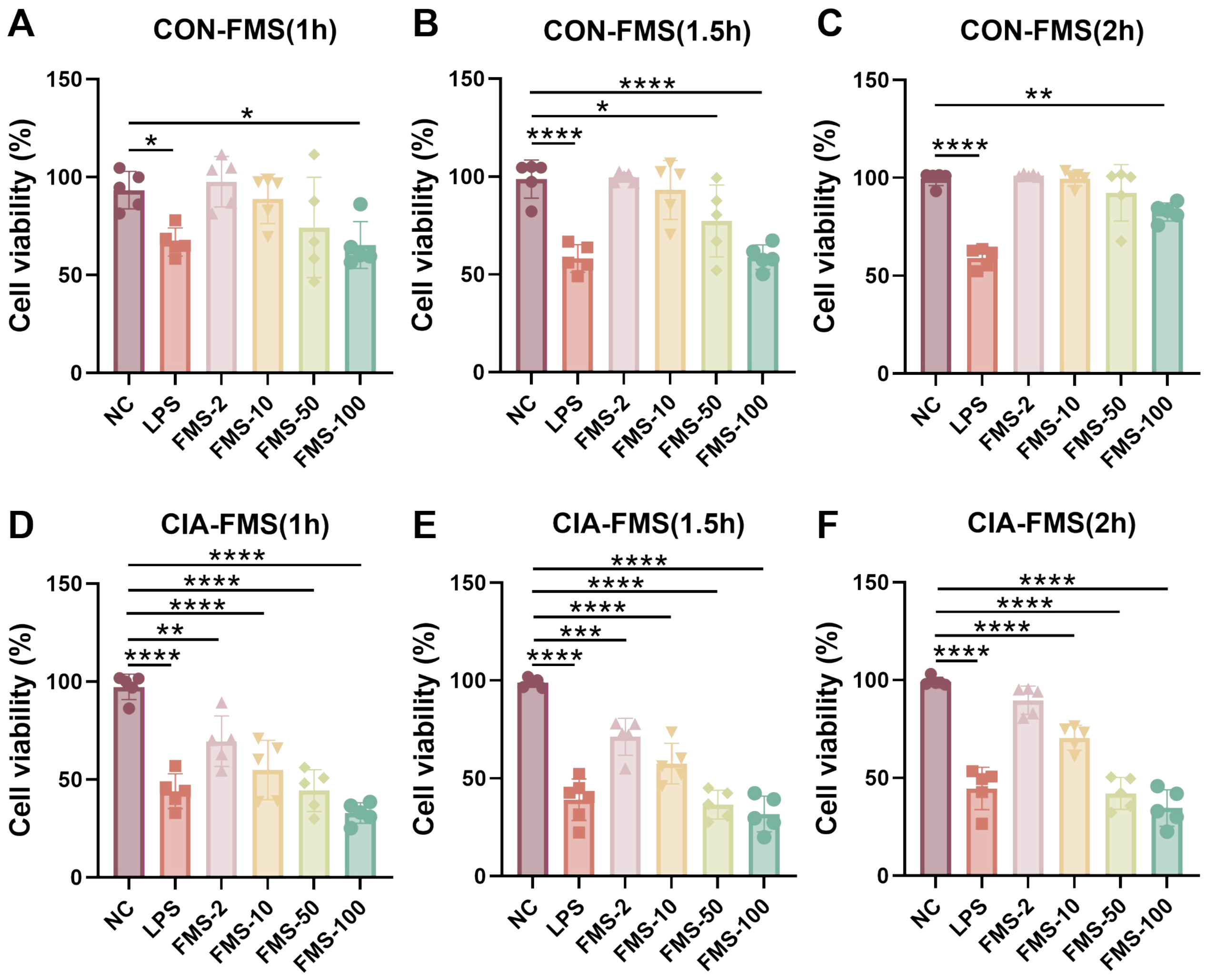

Supplementary Figure S6. CCK-8 results of macrophages stimulated with fecal microbiota supernatant (FMS). FMS from CON rats were incubated with the cells for 1h (A), 1.5h (B) and 2h (C). Meanwhile, cell viability was assessed after the cells stimulated by FMS from CIA animals after 1h (D), 1.5h (E) and 2h (F). The stock solution (FMS-100) was diluted 2- (FMS-50), 10- (FMS-10), and 50- (FMS-2) fold for subsequent experiments. NC: blank control; CON-FMS: FMS from the control group; CIA-FMS: FMS from the CIA group; LPS: lipopolysaccharide. *$P<0.05$, **$P<0.01$, ***$P<0.001$, ****$P<0.0001$ compared with NC group. n=5.

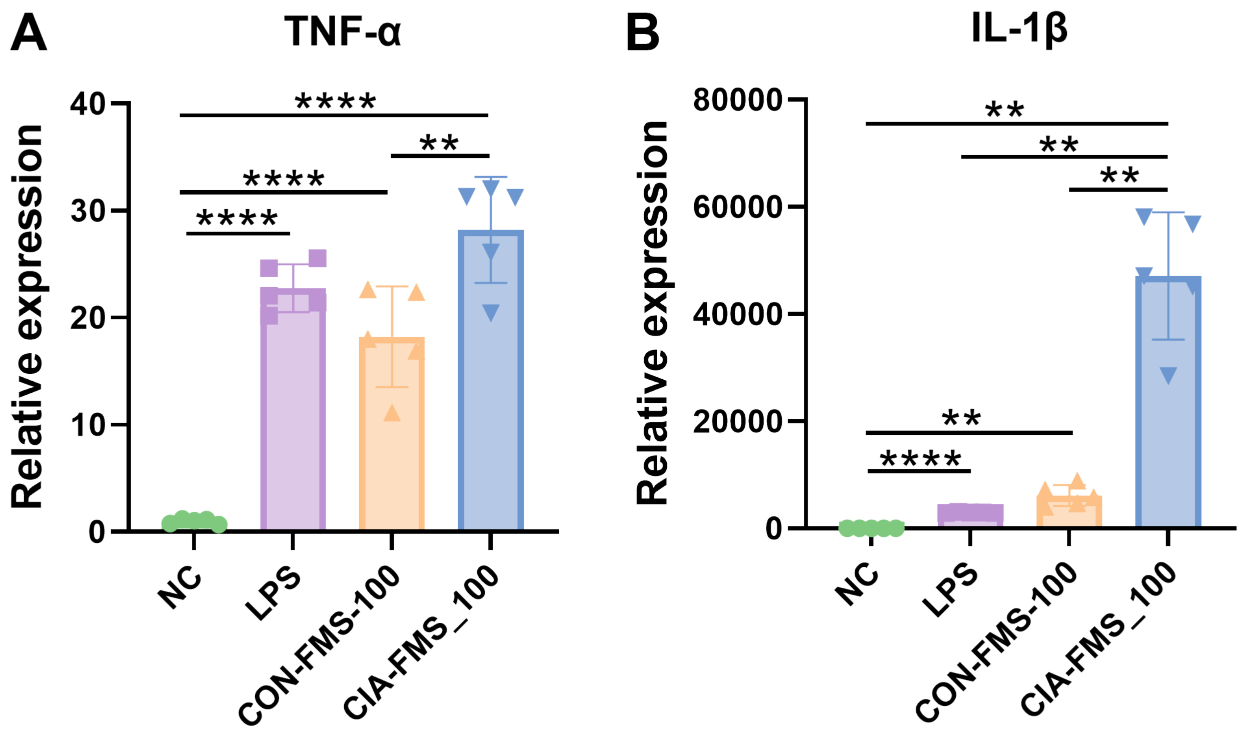

Supplementary Figure S7. qPCR results of TNF-α (A) and IL-1β (B) of macrophages stimulated with LPS and fecal microbiota supernatant (FMS). NC: blank control; LPS: lipopolysaccharide; CON-FMS_100: FMS from the control group; CIA-FMS_100: FMS from the CIA group. ***P*<0.01, *****P* <0.0001 compared with NC group. n=5.

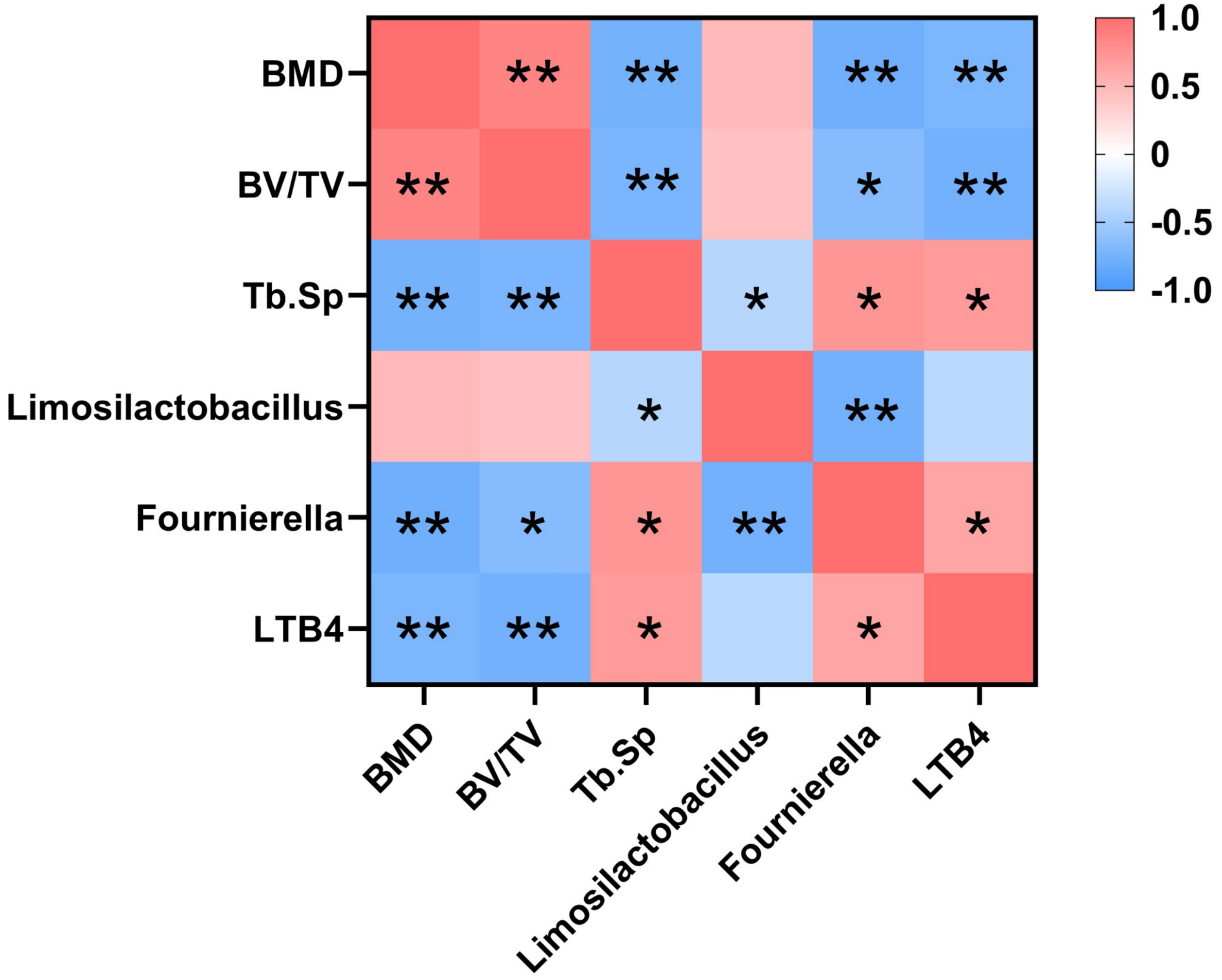

Supplementary Figure S8. Spearman correlation heatmap. Correlation analysis between differentially expressed microbiota, key arachidonic acid metabolites, and bone healing parameters in PBD and CIA+PBD rats at week 3. Each cell represents the Spearman correlation coefficient (r), with significance indicated by *p<0.05 and **p<0.01.
